# High prevalence of somatic *PIK3CA* and *TP53* pathogenic variants in the normal mammary gland tissue of sporadic breast cancer patients revealed by duplex sequencing

**DOI:** 10.1038/s41523-022-00443-9

**Published:** 2022-06-29

**Authors:** Anna Kostecka, Tomasz Nowikiewicz, Paweł Olszewski, Magdalena Koczkowska, Monika Horbacz, Monika Heinzl, Maria Andreou, Renato Salazar, Theresa Mair, Piotr Madanecki, Magdalena Gucwa, Hanna Davies, Jarosław Skokowski, Patrick G. Buckley, Rafał Pęksa, Ewa Śrutek, Łukasz Szylberg, Johan Hartman, Michał Jankowski, Wojciech Zegarski, Irene Tiemann-Boege, Jan P. Dumanski, Arkadiusz Piotrowski

**Affiliations:** 1 Faculty of Pharmacy, Medical University of Gdansk, Gdansk, Poland; 2 3P Medicine Lab, Medical University of Gdansk, Gdansk, Poland; 3 Department of Surgical Oncology, Ludwik Rydygier’s Collegium Medicum UMK, Bydgoszcz, Poland; 4 Department of Breast Cancer and Reconstructive Surgery, Prof. F. Lukaszczyk Oncology Center, Bydgoszcz, Poland; 5 Institute of Biophysics, Johannes Kepler University, Linz, Austria; 6 Department of Immunology, Genetics and Pathology and Science for Life Laboratory, Uppsala University, Uppsala, Sweden; 7 Department of Surgical Oncology, Medical University of Gdansk, Gdansk, Poland; 8 Genuity Science Genomics Centre, Dublin, Ireland; 9 Department of Patomorphology, Medical University of Gdansk, Gdansk, Poland; 10 Department of Tumor Pathology, Prof. F. Lukaszczyk Oncology Center, Bydgoszcz, Poland; 11 Department of Perinatology, Gynaecology and Gynaecologic, Oncology, Collegium Medicum in Bydgoszcz, Nicolaus Copernicus University in Torun, Bydgoszcz, Poland; 12 Department of Oncology and Pathology, Karolinska Institutet, Stockholm, Sweden; 13 Department of Pathology, Karolinska University Hospital, Stockholm, Sweden; 14 MedTech Labs, Bioclinicum, Karolinska University Hospital, Stockholm, Sweden

**Keywords:** Breast cancer, Cancer genetics

## Abstract

The mammary gland undergoes hormonally stimulated cycles of proliferation, lactation, and involution. We hypothesized that these factors increase the mutational burden in glandular tissue and may explain high cancer incidence rate in the general population, and recurrent disease. Hence, we investigated the DNA sequence variants in the normal mammary gland, tumor, and peripheral blood from 52 reportedly sporadic breast cancer patients. Targeted resequencing of 542 cancer-associated genes revealed subclonal somatic pathogenic variants of: *PIK3CA*, *TP53, AKT1*, *MAP3K1*, *CDH1*, *RB1*, *NCOR1*, *MED12*, *CBFB*, *TBX3,* and *TSHR* in the normal mammary gland at considerable allelic frequencies (9 × 10^−2^– 5.2 × 10^−^^1^), indicating clonal expansion. Further evaluation of the frequently damaged *PIK3CA* and *TP53* genes by ultra-sensitive duplex sequencing demonstrated a diversified picture of multiple low-level subclonal (in 10^−2^–10^−4^ alleles) hotspot pathogenic variants. Our results raise a question about the oncogenic potential in non-tumorous mammary gland tissue of breast-conserving surgery patients.

## Introduction

Breast cancer affects 24% of women worldwide and is the leading cause of cancer-related deaths in women^1^. Most breast cancer cases (85–90%) are not associated with inherited mutations of high penetrance genes, such as *BRCA1* (MIM *113705) or *BRCA2* (MIM *600185)^2,3^. High throughput genomics technologies have highlighted the molecular complexity of breast tumors which has led to the molecular classification of four clinically meaningful subtypes: Luminal A, Luminal B, HER2-enriched and basal-like^4,5^. Large cohort studies of breast tumor samples identified somatic driver mutations in key breast cancer-associated genes, such as *PIK3CA* (MIM *171834), *TP53* (MIM *191170), *MAP3K1* (MIM *600982), *CDH1* (MIM *192090), *AKT1* (MIM *164730), *CBFB* (MIM *121360), *TBX3* (MIM *601621), *RB1* (MIM *614041)^6–8^. To date, the identification of somatic driver pathogenic variants has been inferred only from tumors, without providing information on the mutational landscape and allelic frequencies of specific variants in the tissue of cancer origin, i.e., normal tissue of the mammary gland. This is highly relevant as under physiological conditions mammary gland tissue is mitotically stimulated by hormones and undergoes cycles of intense proliferation and remodeling during puberty, pregnancy, and lactation^9^. During life, the mammary gland is exposed to estrogen and its metabolites that damage DNA by single- and double-strand breaks, mutations or, the formation of depurinating adducts^10–12^. These stress conditions can promote the accumulation of post-zygotic, somatic genetic alterations that create the risk of malignant transformation. Indeed, several studies, including ours, have identified such changes in the uninvolved mammary gland of breast cancer patients that is defined as histologically normal glandular tissue, distant from the primary tumor site^13–15^. The most pronounced genetic alterations were identified in the normal tissue from mastectomy patients that per se did not have direct clinical implications, as this affected tissue was removed completely during surgery, but might suggest an increased mutational load in the second breast. At the same time, current clinical management of breast cancer includes breast-conserving surgery (BCS) - removing the tumor and sparing normal breast tissue as one of the recommended treatments^16,17^. The presumed presence of pathogenic genetic alterations in the seemingly normal mammary gland tissue that is not removed during BCS might create a risk of recurrence and can affect future treatment.

Hence, we aimed to screen at unprecedented sensitivity for the presence of subclonal somatic pathogenic genetic alterations in breast cancer-related genes in the normal mammary gland of sporadic cancer patients (study overview in the Supplementary Fig. 1).

Our study demonstrates that structural chromosomal aberrations and clearly pathogenic point variants in crucial breast cancer driver genes are frequent in the normal mammary glandular tissue that remains after breast-conserving surgery.

## Results

### Patterns of chromosomal aberrations

**Fig. 1:**
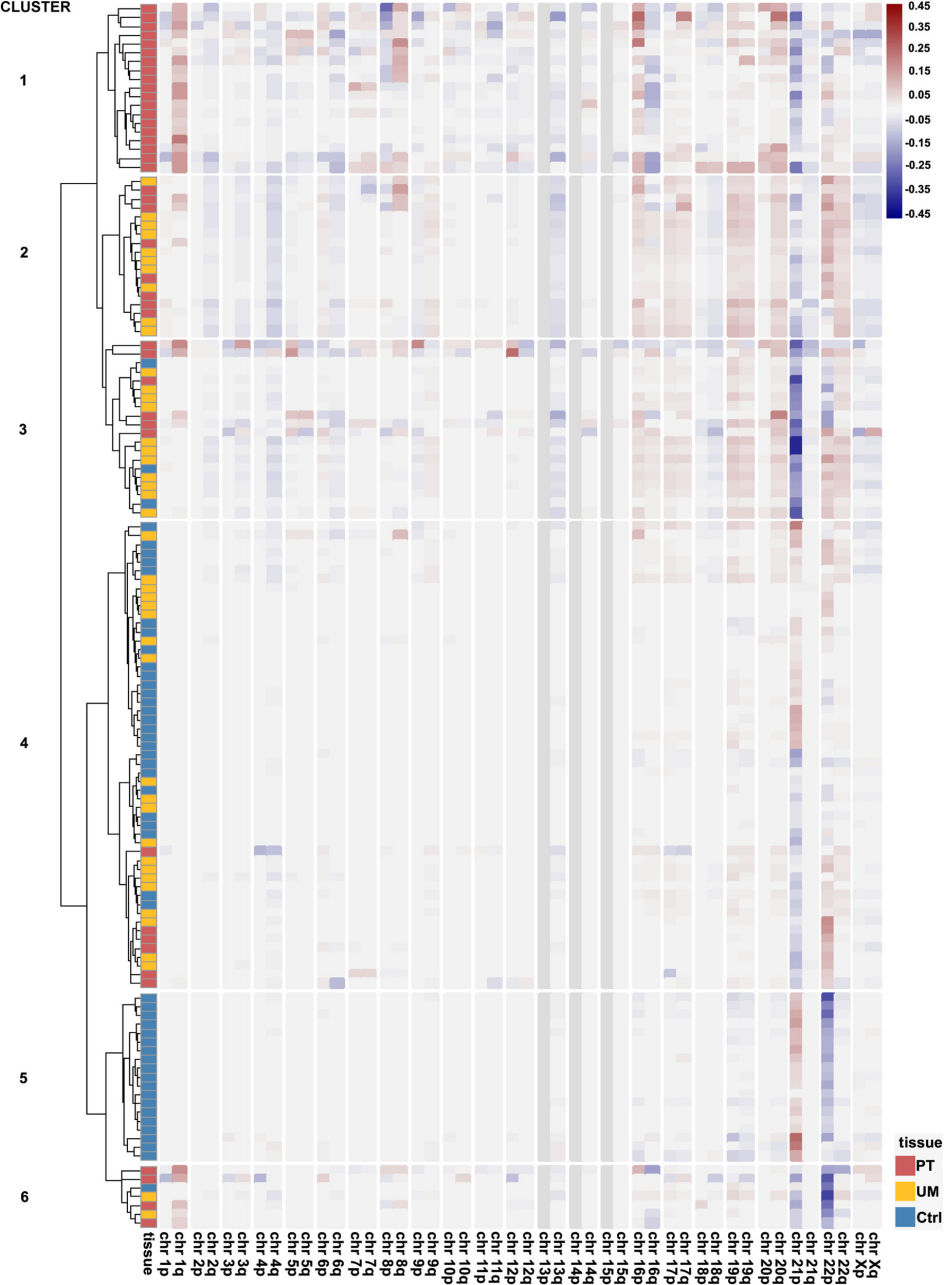
Summary of Copy Number Alterations (CNAs) detected in the studied cohort. Chromosomal CNAs were calculated as mean Log R Ratio (LRR) for chromosome arm and normalized to mean LRR of a sample. Results are presented as a heatmap with colors indicating gains (positive LRR values; red) and deletions (negative LRR values; blue). Hierarchical clustering was performed with Ward2 algorithm^45^ and identified six clusters. Pie charts with proportion of samples within clusters are presented in the Supplementary Fig. 3. Ctrl control cohort mammary gland, UM uninvolved mammary gland, PT tumor.

We carried out analysis of chromosomal rearrangements with SNP arrays to detect DNA copy number alterations (CNAs) as well as copy number neutral loss-of-heterozygosity events via mitotic recombination. In addition to matched samples of normal uninvolved mammary gland (UM) and primary tumor (PT), we included normal mammary gland samples from 26 age-matched women that underwent breast reduction surgery and served as the control group (Supplementary Fig. 2). Spectrum of CNAs in the studied cohort is presented on Fig. 1. Hierarchical clustering revealed two clusters with PT-only and control-only samples and four additional clusters with mixed sample distribution (Supplementary Fig. 3). We also carried out cross analysis of CNAs type, size and number between the studied sample groups. The PTs stand out in this comparison (Wilcoxon test, *p* = 0,0094), with slight differences between normal mammary tissue from breast cancer patients and the control cohort. Nonetheless, per individual basis, total number of CNAs, the number of gains, the size of deletions, and size of CNAs in general were the discriminating features between the normal mammary tissue from breast cancer patients and the control cohort, surprisingly suggesting more heterogeneous nature of the control samples (Supplementary Fig. 4).

We identified recurrent chromosomal aberrations in UMs from sporadic breast cancer patients, such as loss of 1p, 16p11.2, and 9p21.3, and 3q25.3, 4q13.1, 8q, and 20q gains, in line with previous studies^5,18^. Presence of loss of heterozygosity (LOH) at chromosome 8p, associated with poor outcome in breast cancer, was observed in matched UMs and PTs, but also in the normal mammary gland tissue of healthy controls^19^. We observed additional events that frequently accompany 8p LOH, in the UMs: 9p loss and 8q gain. *ERBB2* gains were observed exclusively in PT samples, except for one control mammary gland sample.

### Subclonal somatic pathogenic variants in breast cancer driver genes present in the normal mammary gland tissue

We applied targeted DNA sequencing to identify variants in sets of UM, BL, and PT samples of 52 individuals diagnosed with sporadic breast cancer to distinguish germline and post-zygotic mutations (Supplementary Table 1, Supplementary Table 2).

Four individuals (4/52, 7.7%) were heterozygous for a constitutional pathogenic variant of a known breast cancer-associated gene, i.e. c.5179 A > *T* (p.Lys1727Ter) and c.181 T > *G* (p.Cys61Gly) in the *BRCA1* gene, c.509_510del (p.Arg170fs) and c.354del (p.Thr119fs) in the *PALB2* and *RAD50* genes, respectively (Supplementary Table 3). These results correspond to similar rates from other studies where up to 10% of reportedly sporadic cases turns out hereditary after molecular testing^5,7^. Individuals with germline pathogenic variants were excluded from further analysis, resulting in a total of 48 clearly sporadic breast cancer patients. Constitutional variants of breast cancer-associated genes are listed in the Supplementary Table 3.

**Fig. 2:**
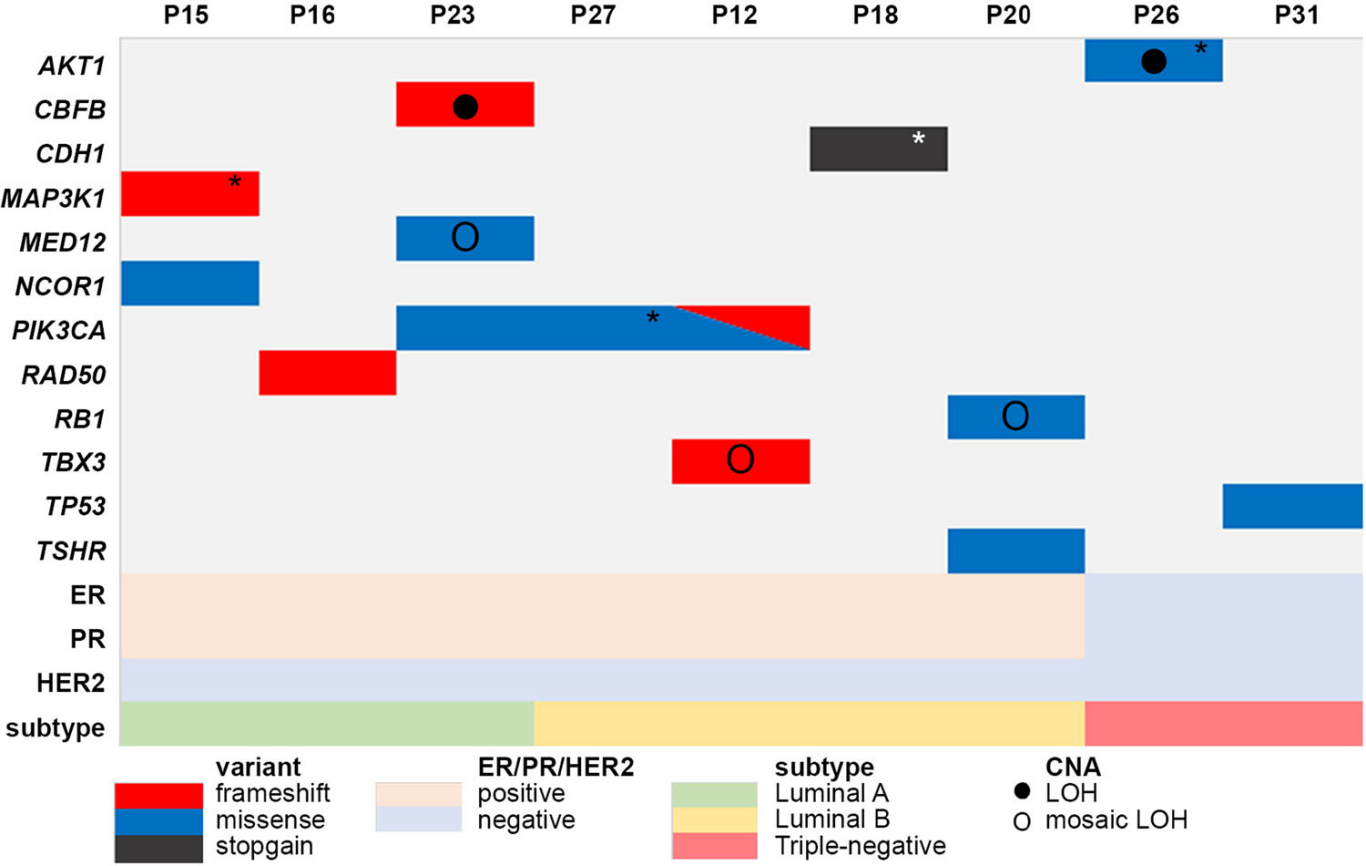
Somatic variants detected in the uninvolved mammary gland (UM). Targeted sequencing revealed somatic variants of known breast cancer-associated genes (rows) present in 9–52% alleles in the UM of sporadic breast cancer patients (columns). Information on estrogen receptor (ER), progesterone receptor (PR), human epidermal growth factor receptor 2 (HER2), and biological subtype of matched primary tumor sample is included. *Variants detected in matched PT sample. CNA Copy Number Alteration status based on SNP arrays. LOH loss of heterozygosity. Description of detected variants, including genomic position and pathogenicity classification is provided in Table 2.

The summary of somatic variants fulfilling the cut-off criteria detected in known breast cancer-associated and candidate breast cancer-associated genes is provided in Supplementary Tables 4 and 5, respectively. We identified 15 somatic pathogenic, likely pathogenic variants or variants of uncertain significance with predicted deleterious effect on the encoded protein in the normal mammary gland tissue of 19% (9/48) of patients (Fig. 2). The affected genes are tumor suppressors (*TP53*^5^*, RB1*^20^*, CDH1*^21^), oncogenes (*PIK3CA*^22^), regulate cell death (*MAP3K1*^23^), DNA repair (*AKT1*^24^*, RAD50*^25^), translation (*CBFB*^26^), gene expression (*MED12*^27^*, TSHR*^28^) and chromatin remodeling (*NCOR1*^6^). A detailed description of these genes in the context of breast cancer is provided in Supplementary Tables 6, 7 and Supplementary Fig. 8. All of these variants except *PIK3CA* c.3140 A > G (p.His1047Arg) were detected in BCS patients, in samples from the tissue portion that was not qualified for surgical resection.

### Heterogeneity of *PIK3CA* and *TP53* pathogenic variants revealed in the normal mammary gland tissue

Two driver genes dominate across all subtypes of invasive breast cancer: *PIK3CA* and *TP53*^5^. *PIK3CA* encodes the catalytically active p100alpha isoform that regulates cell proliferation and growth receptor signaling cascade. Activating *PIK3CA* point variants are the most prevalent in breast tumors and were confirmed to lead to malignant transformation^22,29^. We detected four hotspot *PIK3CA* somatic variants in the uninvolved mammary gland, all of them have been described in the COSMIC database and reported in breast tumors (Fig. 2, Table 2, Supplementary Fig. 5). *TP53* tumor suppressor acts as a transcription factor and is frequently inactivated in human malignancies, mostly through loss-of-function *TP53* variants^30–32^. We detected an Ile195Thr hotspot variant in the uninvolved mammary gland that affects the central DNA-binding domain (Fig. 2, Table 2, Supplementary Fig. 5).

**Fig. 3:**
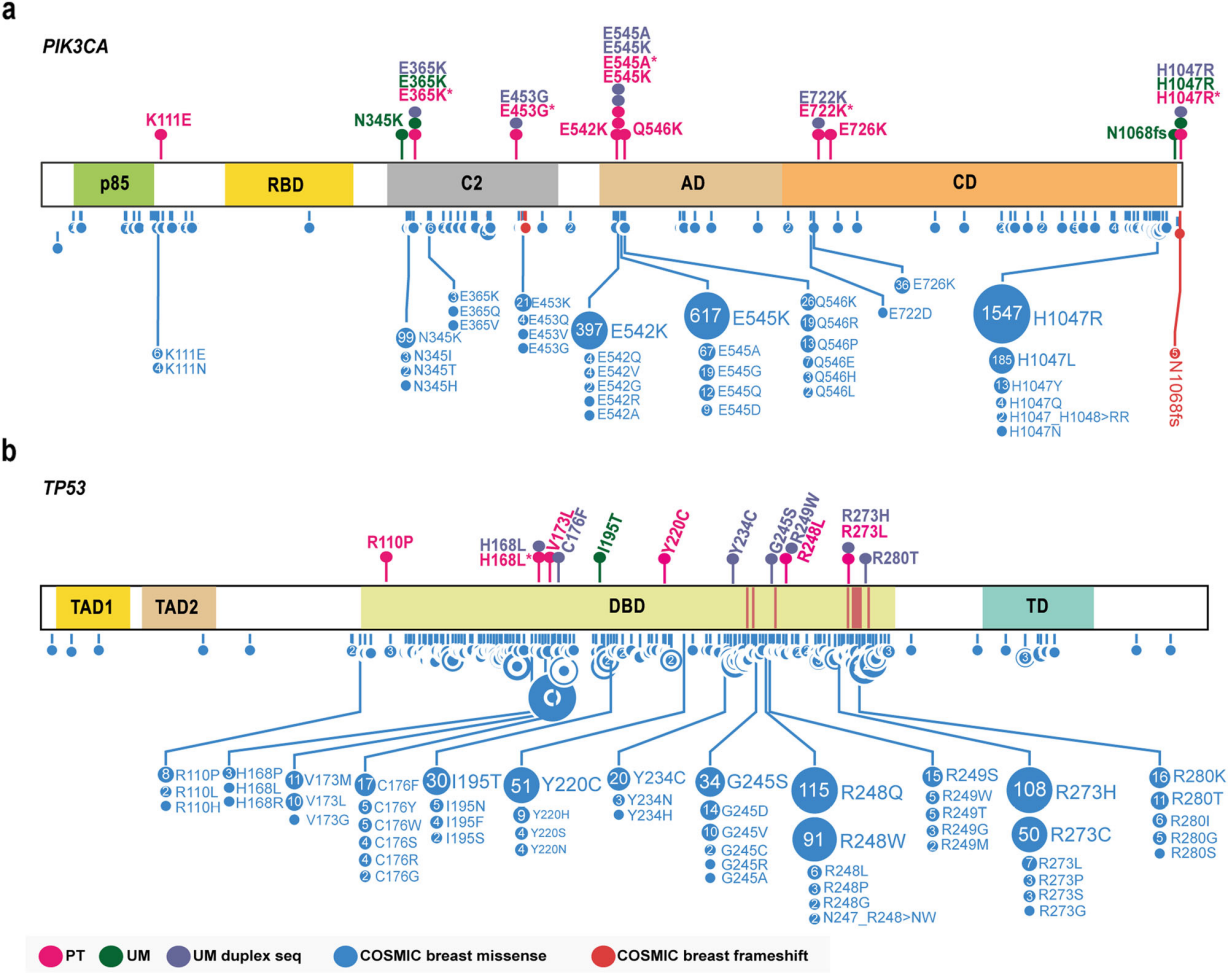
Somatic *PIK3CA* and *TP53* variants detected in the uninvolved mammary gland (UM) and primary tumor (PT) samples. Lollipop plots represent somatic variants of (**a**) *PIK3CA* and (**b**) *TP53* genes detected by targeted next-generation sequencing (NGS). Upper panel represents variants detected in patient uninvolved mammary gland (UM) and tumor (PT) samples. All somatic variants detected according to the standard NGS and pathogenic/likely pathogenic variants detected by duplex sequencing in UM samples are included. Lower panel is a summary of somatic variants detected in breast tumors reported in the COSMIC database (https://cancer.sanger.ac.uk/cosmic). p85 p85-binding domain, RBD Ras-binding domain, C2 C2 domain, AD accessory domain, CD catalytic domain. TAD1, TAD2 transcription activation domain 1 and 2, DBD DNA-binding domain, DNA-binding sites are marked with red lines, TD tetramerization domain. Lollipop plots were prepared based on the images generated with the Protein paint application^52^. *Variants detected by standard NGS in primary tumor samples and selected for duplex sequencing.

To enhance the sensitivity and accuracy of rare variant detection, we employed duplex sequencing (Supplementary Fig. 7). We selected four individuals: P10, P28, P51, and P52 based on the presence of *PIK3CA* and *TP53* hotspot variants in PT samples according to standard NGS data (Fig. 3) and screened for variants in the normal mammary gland samples with high sensitivity duplex NGS sequencing. Ultra-deep targeted duplex sequencing of *PIK3CA* detected low-level subclonal pathogenic variants: c.1093 G > A (p.Glu365Lys), c.1358 A > G (p.Glu453Gly), c.1633G > A (p.Glu545Lys) c.1634A > C (p.Glu545Ala), c.2164 G > A (p.Glu722Lys), c.3140 A > G (p.His1047Arg), in the uninvolved mammary gland samples of three individuals. The detected variants were located in the known *PIK3CA* hotspot regions, reported in breast tumors in the COSMIC database and functionally confirmed to affect PIK3CA function^7,22^ (Fig. 3, Supplementary Table 8). A screen for *TP53* variants not only confirmed the presence of His168Leu variant, but also revealed additional hotspot variants: c.527 G > T (p.Cys176Phe), c.701 A > G (p.Tyr234Cys), c.733 G > A (p.Gly245Ser), c.745 A > T (p.Arg249Trp), c.818 G > A (p.Arg273His), c.839 G > C (p.Arg280Thr). Importantly, all these pathogenic variants are located in the central DNA-binding domain indispensable for p53 tumor-suppressive function^7,32^ (Fig. 3, Supplementary Table 8).

## Discussion

**Fig. 4:**
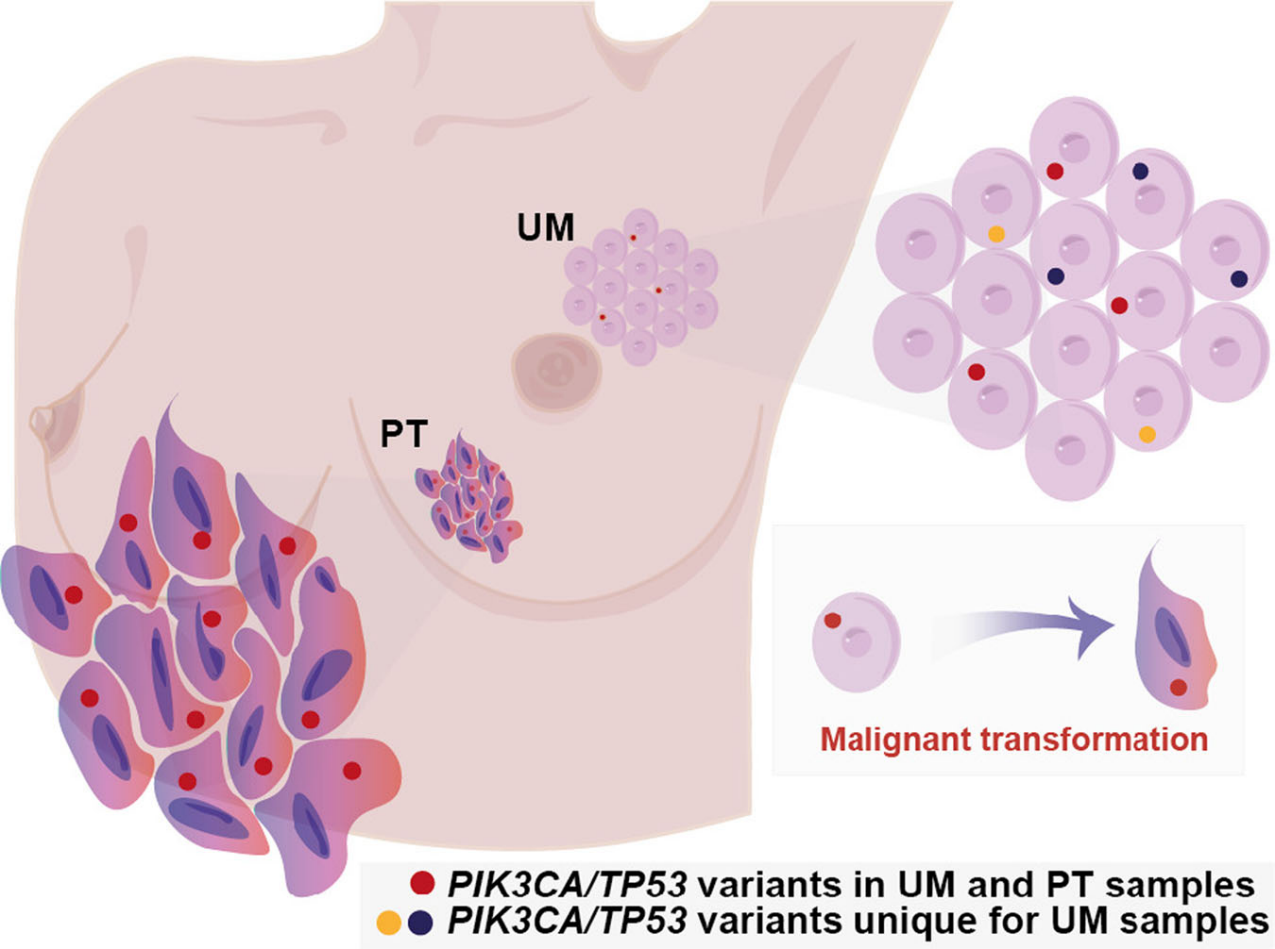
Oncogenic potential of the normal mammary tissue. We used duplex sequencing to screen for ultra-low frequency variants and detected *PIK3CA* and *TP53* hotspot alterations. The sampled normal mammary gland tissue is referred to as uninvolved glandular tissue and was not removed during surgical resection of the tumor mass. Detected variants might alter the function of the main breast cancer drivers: activate *PIK3CA* oncogene and impair *TP53* tumor suppressor DNA-binding capacity. The presence of these changes implicates an oncogenic potential of the uninvolved mammary gland tissue and emphasizes the importance of thorough monitoring of sporadic breast cancer patients that underwent breast-conserving surgery.

Post-zygotic variations contribute to the genetic heterogeneity of an individual, which is reflected in a mosaic pattern of genetic alterations in all cells that make up the human body^33^. The mammary gland remains mitotically active during life and under physiological conditions is exposed to DNA-damaging estrogen metabolites^11^. Subclonal somatic genetic changes acquired during life pose a risk of cancer development. Hence, we hypothesized that these factors can increase the mutational burden in the mammary gland. Other studies have reported the presence of genomic and transcriptomic changes in the normal mammary gland, and suggested that histological normalcy does not exclude pathological biological changes^34–36^. However, these studies have been carried out on normal mammary tissue obtained from mastectomies or cancer-adjacent samples, hence the clinical relevance of the these findings was limited. In this study, we screened for somatic genetic changes in the normal mammary gland tissue of sporadic cancer patients, including tissue biopsies from the parts of the breast that normally would not have been removed during breast-conserving surgery. We identified widespread genomic structural rearrangements that affect gene dosage and somatic subclonal sequence variants of known breast cancer-associated genes that control proliferation, cell death, metastasis, and genome integrity: *PIK3CA*, *TP53, AKT1*, *MAP3K1*, *CDH1*, *RB1*, *NCOR1*, *MED12*, *CBFB*, *TBX3*, and *TSHR* (Supplementary Fig. 8). These variants were present in a considerable percentage of cells, suggesting they occurred earlier in the mammary gland development or the carrier cells gained growth advantage and underwent clonal expansion. Further, ultra-sensitive duplex sequencing revealed heterogenous mosaic landscape of low-level subclonal pathogenic variants of main breast cancer drivers: *PIK3CA* and *TP53* in the normal mammary gland tissue. Notably, the setup of these variants was markedly different between tumor and normal mammary tissue from the same individuals which is suggestive of multiple, independent mutational events that occurred in the mammary gland (Fig. 4).

In parallel to sequence variants, we identified recurrent CNAs in the mammary gland of breast cancer patients, but also in the age-matched control group (Fig. 1). This facilitated detecting subtle, but noticeable differences in terms of total number and length of all detected CNAs per individual (Supplementary Fig. 4). Both groups: breast cancer and control were age-matched and therefore the mammary gland tissue was exposed to cycles of estrogen for comparable time and that can explain the accumulation of copy number alterations in both cohorts.

The most important finding from this part of our study is that the normal mammary tissue from cancer patients showed DNA copy number alterations as well as evidence of copy number neutral loss-of-heterozygosity. These genomic alterations in concert with damaging sequence variants recapitulate alternative routes of gene inactivation that are typically observed in the malignant tumors, but not in the benign tissue. In this context, our study demonstrates that normal tissue profiling provides direct information on the very origin of the disease and may improve the choice of treatment as well as may aid in further clinical management of the affected individuals^37–39^. This is in contrast to typical molecular profiling studies that rely on limited retrospective information inferred from the tumors.

The *PIK3CA* and *TP53* genes are the leading oncogenic mutations of breast malignancies and accordingly the most common changes detected in our study were in the *PIK3CA* gene^5,40^. Soysal et al. screened for somatic variants in benign biopsies of patients that subsequently developed breast cancer. *PIK3CA* and *TP53* variants were the most prevalent changes in tumor samples, but not detected in benign biopsies, possibly due to limited sensitivity of standard massively parallel sequencing for rare variant detection^41^. To overcome this limitation, we implemented duplex sequencing technology to detect *PIK3CA* and *TP53* variants in the normal mammary gland samples at very low frequency. In the uninvolved mammary gland tissue, we detected known hotspot pathogenic variants that might activate *PIK3CA* kinase or target DNA-binding domain of *TP53* tumor suppressor, disabling its function.

We confirmed that these variants observed in tumor samples were already present in the normal glandular tissue as well, albeit at lower levels compared to the corresponding tumors. Strikingly these changes were accompanied in the same samples by other *PIK3CA* and *TP53* pathogenic variants, present in the normal tissue, but not in the corresponding tumors. This may suggest the existence of potential sites of secondary tumor formation. Notably, the majority of somatic pathogenic variants, including these *PIK3CA* and *TP53* hotspot alterations, occurred in the normal mammary gland samples not removed during breast-conserving surgery, not from radical mastectomy patients.

At the same time *PIK3CA* and *TP53* variant spectra in the normal glandular tissue were more similar to the ones reported in cancer-oriented database (COSMIC) than those in general population (gnomAD), suggesting that the studied UM tissues reflect the repertoire of somatic variants seen in tumor samples (Supplementary Fig. 9, Supplementary Fig. 10, Supplementary Table 9). However, given the limited number of four individuals included in duplex sequencing analysis, these conclusions should be interpreted with caution. Further studies on a larger well-characterized cohort of sporadic breast cancer patients are needed for understanding how specific variants arise and expand during life. Nevertheless, we demonstrate here that ultra-sensitive duplex sequencing approach might be beneficial to detect very low-level frequency somatic mosaicism in different tissue samples, with its potential clinical implications in terms of molecular diagnostics and prognosis.

After surgical intervention, breast cancer patients remain under clinical surveillance with recommended yearly mammogram and physical examination every 3–4 months for the first two years after surgery^42^. The current diagnostic approach has been focused mainly on the identification of constitutional pathogenic variants in known breast cancer-associated genes to catch early these individuals who are in a higher risk of breast cancer development and/or to whom the personalized targeted therapy could be offered. However, over 80% of all breast cancer cases are not associated with inherited changes^17^.

Our results demonstrate a complex landscape of mutational burden in the seemingly normal mammary glandular tissue and indicate an oncogenic potential of the tissue not removed during surgery. This study provides a rationale for thorough genetic and clinical surveillance of sporadic breast cancer patients that underwent breast-conserving surgery. Including molecular evaluation of the normal glandular tissue of sporadic breast cancer patients could be beneficial for personalized patient care.

## Methods

### Patient samples and DNA isolation

We analyzed samples from 52 patients diagnosed with reportedly sporadic breast cancer with an emphasis on breast-conserving surgery (2/3 of the patients studied) and who did not receive neoadjuvant therapy. Altogether a total of 204 uninvolved mammary gland (UM), primary tumor (PT), skin (SK), and peripheral blood (BL) samples were collected via the Oncology Centre in Bydgoszcz and the University Clinical Centre in Gdansk, with the approval of bioethics committee at Medical University of Gdansk (MUG). We have obtained written informed consent from all participants. PT, UM, SK, and BL samples from each patient were collected and stored in −80 °C upon DNA isolation. The overview of sample processing workflow is presented in the Supplementary Fig. 1. The histological subtypes and tumor tissue content of each PT sample were evaluated by pathologists according to the current American Joint Committee on Cancer guidelines^43^. Tumor samples with less than 50% of neoplastic cell content were excluded. The normal mammary gland was sampled preferably from the opposite quadrant relative to the primary tumor site, with a mandatory cut-off criterion of at least 3 cm in each case, to exclude potential contamination with residual tumor cells. These tissue samples were also evaluated by pathologists to confirm normal histology (Table 1, Supplementary Table 1). All normal mammary gland samples from patients who underwent breast-conserving surgery were derived from the portion of tissue that remained intact in the patient body after breast-conserving surgery. Solid tissues were homogenized in a lysis buffer, then Proteinase K was added and samples were incubated at 55 °C for 48 h. DNA isolation from UM, PT, and SK tissue lysates was performed by phenol–chloroform extraction as previously described^13^. Blood DNA extraction was performed with the QIAamp DNA Blood Mini Kit according to the manufacturer’s protocol (Qiagen, Germantown, MD).

**Table 1:** Summarized clinicopathological features of sporadic breast cancer patient cohort.

Number of individuals	52
**Collected samples:**	204
UM	52
PT	52
BL	52
SK	48
**Age (median/range)**	45/28–60
**Histology**	
IDC	44
ILC	4
IDC-ILC	1
other	3
**Receptors**	
ER (positive/negative)	46/6
PR (positive/negative)	46/6
HER2 (positive/negative)	5/47
**Subtype**	
Luminal A	22
Luminal B	24
HER2-enriched	2
Triple-negative	4

Uninvolved mammary gland tissue (UM), primary tumor (PT), skin (SK), and peripheral blood (BL) samples were collected from 52 individuals diagnosed with reportedly sporadic breast cancer. Histological evaluation of tumor samples was performed according to the current American Joint Committee on Cancer guidelines^43^. PT samples were classified as Invasive Ductal Carcinoma (IDC), Invasive Lobular Carcinoma (ILC), mixed (ICD-ILC) or other. Estrogen (ER), progesterone (PR), and ERBB2 (HER2) receptors were evaluated based on immunostaining or immunostaining and FISH (HER2). Biological subtypes were assigned based on ER/PR/HER2 and Ki67 status. Detailed clinicopathological information is provided in the Supplementary Table 1.

### Copy number alteration detection

SNP array genotyping was performed for UM and PT samples on an Illumina Infinium Global Screening Array, according to the manufacturer’s recommendations (Illumina, San Diego, CA). SNP genotyping data from mammary gland tissues of 26 age-matched women that underwent breast reduction surgery were used as control samples (Supplementary Fig. 2). Genotyping data was analyzed using Nexus Copy Number software version 10.0 (BioDiscovery). Quality control of samples was performed as described previously^14,44^. Briefly, samples with Log R Ratio (LRR) sd > 0.2 were flagged as poor quality and excluded from the analysis. The analysis was performed with default settings except that significance threshold for Copy Number Alterations (CNA) calling was decreased to 5*10^−13^- (default 5*10^−7^), minimal number of probes per segment was increased to 10 (default 3), gain threshold was set to 0.49 and 0.14 which corresponds to approximately 40% and 10% change for a high gain and gain respectively (the default is 0.41 and 0.06 for a high gain and gain), the loss threshold was set to −0.16 and −0.74 what corresponds to approximately −10% and −40% change for a loss and high loss respectively (the default is −0.09 and −1.1 for a loss and high loss). Hierarchical clustering was performed using the Ward2 algorithm^45^.

### Statistical analysis

All statistical analyses were carried out using R version 3.6.2 and package *stats*. Packages *pheatmap* and *ggpubr* were used for plotting. Statistical significance of differences between two groups was tested using the Mann–Whitney U test. Differences were considered significant at a two-sided *p* < 0.05.

### Targeted DNA resequencing

Targeted DNA sequencing panel was designed with Roche NimbleDesign online tool (Roche, https://hyperdesign.com/). The panel included exons with + /- 50 kbp flanking regions of 542 genes selected based on in-house database and literature research (Supplementary Table 2). Sequencing libraries were prepared for sets of UM, BL, and PT samples with the capture-based Roche SeqCap EZ system according to the manufacturer’s protocol (Roche, Pleasanton, CA), followed by 150 bp paired-end sequencing performed on Illumina NextSeq550 and MiniSeq instruments (Illumina, San Diego, CA). Sequencing read alignment to the human reference genome (hg38) was performed with the Burrows–Wheeler transform aligner (http://bio-bwa.sourceforge.net/)^46^. Platypus v.0.8.1.1 (https://www.rdm.ox.ac.uk/research/lunter-group/lunter-group/) was used for variant calling^47^. Variants with poor mapping quality (<30), variants supported by high-quality bases (≥30) in fewer than five reads, and variants outside the targeted regions were excluded from analysis. Variants were annotated with VarAFT (version 2.17-2) software^48^.

For variant selection, only variants with sequencing depth ≥ 30 and tissue allele frequency ≥ 0.07 were included in the analysis. All truncating variants were included. For non-truncating variants, the following criteria were used: variants were filtered by their clinical significance as reported in the ClinVar database (as of June 2021), variants classified as Pathogenic, Likely Pathogenic, Conflicting interpretations of pathogenicity, risk factor, and drug response were included in the study. The remaining non-truncating variants were included based on their frequency in the general population: variants with minor allele frequency (MAF) ≤ 0.001 across all gnomAD populations (“popmax”) or not noted in the database were included. For in silico splicing analysis splice prediction algorithms, i.e. SSF, MaxEntScan, and NNSplice, embedded in Alamut Visual software (version 2.14) were used. Variants described in this study were classified according to the American College of Medical Genetics and Genomics and the Association for Molecular Pathology recommendations^49^. Based on literature^2,7,30,50,51^ we selected 155 breast cancer-associated genes that were the primary focus of variant analysis (Supplementary Table 2). Somatic variants presented in Fig. 2 and Table 2 were confirmed by Sanger sequencing or High Resolution Melting analysis (Supplementary Fig. 5). Lollipop plots with variant demonstration were prepared based on images generated with the Protein paint application^52^.

**Table 2:** Pathogenicity classification of somatic variants detected in the uninvolved mammary gland (UM) samples.

ID	Gene	Genomic position^a^	cDNA change (protein change)^b^	ACMG classification^c^	rsID^d^	ClinVar^e^	UM allele frequency^f^	PT allele frequency^f^
**P26**	*AKT1*	chr14:104780214	c.49 G > A (p.Glu17Lys)	Pathogenic	rs121434592	–	0,11	0,36
**P23**	*CBFB*	chr16:67036674	c.207dup (p.Pro70fs)	Pathogenic	–	–	0,15	not detected
**P18**	*CDH1*	chr16:68819382	c.1668_1669insT (p.Lys557Ter)	Pathogenic	–	–	0,1	0,17
**P15**	*MAP3K1*	chr5:56881868	c.2668del (p.Asn891fs)	Pathogenic	–	–	0,09	0,15
**P23**	*MED12*	chrX:71137882	c.5983 C > T (p.Pro1995Ser)	Likely pathogenic	–	–	0,15	not detected
**P15**	*NCOR1*	chr17:16040459	c.6715 C > A (p.Pro2239Thr)	Likely pathogenic	–	–	0,11	not detected
**P12**	*PIK3CA*	chr3:179234358	c.3203dup (p.Asn1068fs)	Pathogenic	rs587776802	Pathogenic	0,19	no data^g^
**P12**	*PIK3CA*	chr3:179204536	c.1093 G > A (p.Glu365Lys)	Pathogenic	rs1064793732	Pathogenic	0,33	no data^g^
**P23**	*PIK3CA*	chr3:179203765	c.1035 T > A (p.Asn345Lys)	Pathogenic	rs121913284	Likely pathogenic	0,11	not detected
**P27**	*PIK3CA*	chr3:179234297	c.3140 A > G (p.His1047Arg)	Pathogenic	rs121913279	Pathogenic	0,11	0,11
**P16**	*RAD50*	chr5:132595759	c.2165dup (p.Glu723fs)	Pathogenic	rs397507178	Pathogenic	0,16	not detected
**P20**	*RB1*	chr13:48345117	c.418 A > G (p.Thr140Ala)	Likely pathogenic	–	–	0,11	not detected
**P12**	*TBX3*	chr12:114679572	c.796_797dup (p.Ser266fs)	Pathogenic	–	–	0,18	not detected
**P31**	*TP53*	chr17:7674947	c.584 T > C (p.Ile195Thr)	Pathogenic	rs760043106	Likely pathogenic	0,52	no data^g^
**P20**	*TSHR*	chr14:81068264	c.253 A > G (p.Ile85Val)	VUS	–	–	0,13	not detected

Targeted DNA sequencing identified somatic DNA variants of known breast cancer-associated genes in the uninvolved mammary gland tissue of sporadic breast cancer patients.

^a^ Genomic position according to the hg38 sequence assembly.

^b^ Variant annotation provided for the basic isoform of the transcript.

^c^ Pathogenicity classification according to the current ACMG guidelines^49^.

^d^ rsIDs in dbSNP build 152.

^e^ Variant pathogenicity classification according to the ClinVar database. Detailed description of somatic variants detected in UM samples is provided in the Supplementary Table 4.

^f^ Tissue allele frequency of the detected variants in matched UM and PT tissue specimens.

^g^ PT sample was not available.

Confirmation of somatic variants by Sanger sequencing or high-resolution melting is provided in the Supplementary Fig. 5. VUS Variant of Unknown Significance.

### Duplex sequencing

UM, PT, BL, and SK samples of four individuals (P10, P28, P51, and P52) were selected for detection of variants by duplex sequencing based on the presence of *PIK3CA* or *TP53* hotspot variants in PT, but not UM tissue, according to standard NGS. The protocols used here are based on the ones described in more detail in Salazar et al.^53^.

#### Random DNA shearing and size selection

DNA was ultrasonicated for 10 min at ≤10 °C using a Bandelin Sonorex Super RK 102 H Ultrasonic bath ending up with a fragment size distribution of, on average, 275 bp. A double-size selection was performed using Sera-Mag Select beads (Cytiva) in order to exclude fragments outside a range of 100-400 bp. The size selection was performed in 50 µl of sonicated DNA (2 µg), 20 µl 10x CutSmart buffer (NEB), 47.6 µl PCR grade water with 0.7 volumes beads. The reaction was mixed by pipetting thoroughly and incubated at room temperature (RT) for 10 min. Tubes were then placed on a magnet for 5 min and 190 µl of supernatant was transferred to a fresh tube. Next, 2.5 volumes of beads in total considering the initial bead solution was added to the solution and mixed by pipetting. The mixture was incubated at RT for 10 min. Tubes were placed on a magnet and supernatant was discarded. The beads were washed twice with 80% ethanol, air dried at room temperature and 23 µl of PCR grade water was added to resuspend by pipetting. After incubating at RT for 5 min, the dissolved beads were allowed to stand at RT for 5 min, placed on a magnet and the clear supernatant containing the size-selected DNA was transferred to a new tube.

#### End-repair, A-tailing, adapter ligation, and bead purification

Size selected genomic DNA was end-repaired and A-tailed using the NEBNext® Ultra™ II End Repair/dA-Tailing Module (New England Biolabs) according to the manufacturer’s instructions followed by adapter ligation with the NEBNext® Ultra™ II Ligation Module (New England Biolabs) following the manufacturer’s instructions. The adapters ligated to the A-tailed DNA were synthesized as previously described (Adapter 2)^53^. The ligation reaction was then purified using 1.2 volumes of Sera-Mag Select beads (Cytiva). A total of 96.5 µl sample was thoroughly mixed with 115.8 µl beads by pipetting and incubated at RT for 10 min. Tubes were placed on a magnet and the supernatant was discarded. The beads were washed twice with 80% ethanol. Next, the beads were dried at room temperature and 23 µl of PCR grade water was added to resuspend by pipetting. After incubating the dissolved beads at RT for 5 min they were placed on a magnet and the clear supernatant containing the purified DNA was transferred to a fresh tube.

#### Pre-capture amplification

Ligated fragments were amplified with KAPA HiFi HotStart ReadyMix PCR Kit (KAPA Biosystems). Reaction components, primer sequences, and cycling conditions are listed in the Supplementary Table 10. For libraries with input DNA higher than 240 ng, two parallel reactions were prepared and pooled in the end, just before purification. The first step of amplification was 6 or 12 cycles of single primer extensions followed by the addition of the primer NEBNext Universal and a standard PCR amplification of 2 cycles. PCR products were purified with 1.2 volumes Sera-Mag Select beads as described above, followed by two rounds of targeted capture steps to enrich the templates of interest.

#### Targeted captures and post-capture amplification

Two rounds of targeted captures followed by PCR amplification were performed as described in Salazar et al., with minor modifications on the post-capture amplification (Supplementary Table 10)^53^. The biotinylated probes used to target exonic regions of *TP53*, and *PIK3CA* are detailed on Supplementary Table 10.

### Duplex sequencing data analysis

FastQ files were analyzed with Galaxy platform (available on a private server provided by the Medical University of Gdansk) and first processed by the tool *Trim Galore!* to trim Illumina-specific adapter sequences including the barcode and spacer sequence at the 3' end of the raw reads. Next, the reads were analyzed according to a duplex sequencing (DS) specific pipeline that includes an error correction tool^54^. After creating the duplex consensus sequence (DCS), a trimming step of 5 nucleotides from both 5' and 3' end was included. The trimmed consensus sequences were then aligned by *BWA-MEM* and *BamLeftAlignIndels* to the human genome assembly hg38. To avoid false-positive variants that would occur within any partial adapter sequences and barcodes at the 3' end of the consensus sequence and were not removed by the first adapter trimming step, the tool *clipOverlap* from the package BamUtil was applied. Variant calling was then performed by the variant caller *LoFreq*. Finally, the variants (substitutions only) were further inspected and assigned to tiers using the *Variant Analyzer*^55^. Variants with DCS coverage below 500 and variants outside the probe regions were discarded from our analysis and only Tier 1 variants were kept, together with Tier 2 that were detected more than once. For more details on this analysis see Povysil et al.^55^. The full Galaxy workflow is publicly available: https://usegalaxy.org/u/jku-itb-lab/w/gdansk-paper---galaxy-workflow.

The variant frequency was calculated by dividing the number of DCS calling the variant by the DCS coverage at the position of the variant within the library it was detected. The variant frequency was calculated by the count for each alteration type (e.g. A > C) divided by the frequency of the sequenced reference allele (e.g., frequency of A’s in the reference sequence multiplied by the sum of the mean DCS coverage for that library). The relative count is the count for each variant type divided by the sum of all occurring variants within the tissue.

## Supplementary information

Supplementary Information

Supplementary Table 1

Supplementary Table 2

Supplementary Table 3

Supplementary Table 4

Supplementary Table 5

Supplementary Table 6

Supplementary Table 7

Supplementary Table 8

Supplementary Table 9

Supplementary Table 10

## Data Availability

Raw microarray, NGS and duplex sequencing data are available upon request in the EGA archive, study ID EGAS00001005698.
