# Supplementary material for: High prevalence of somatic PIK3CA and TP53 pathogenic variants in the normal mammary gland tissue of sporadic breast cancer patients revealed by duplex sequencing
Source: NPJ Breast Cancer. 2022 Jun 29;8:76. doi: 10.1038/s41523-022-00443-9 (PMC9243094; doi:10.1038/s41523-022-00443-9)
Supplement: Supplementary file 1 — Supplementary Information [file 41523_2022_443_MOESM1_ESM.pdf]

Supplementary Figure 1

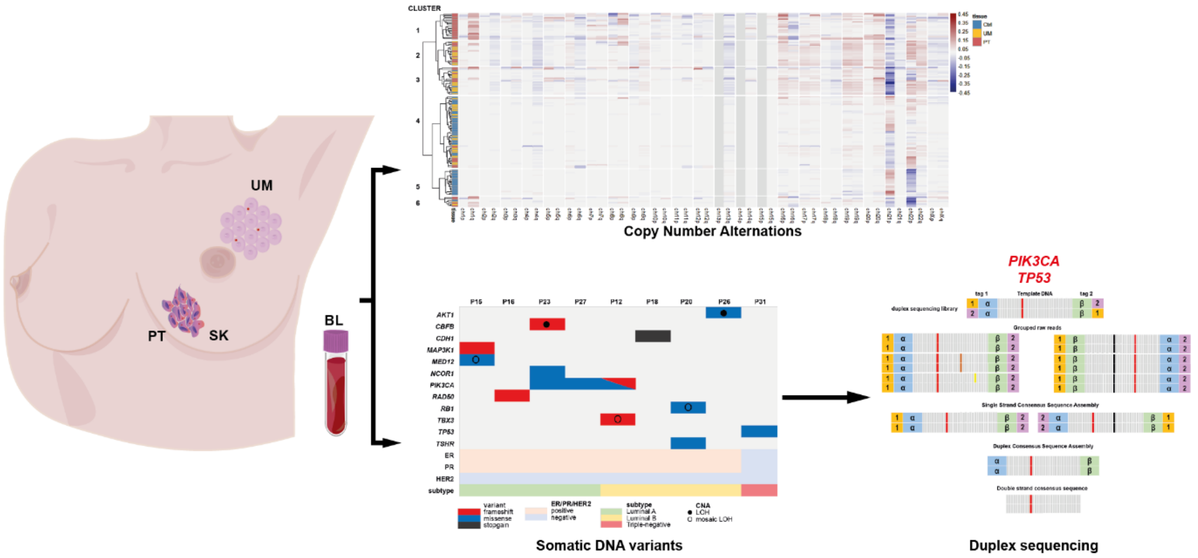

Supplementary Figure 1. Graphical representation of the project workflow. A total of 204 fresh-frozen uninvolved mammary gland (UM), primary tumor (PT), skin (SK) and peripheral blood (BL) samples were collected from 52 individuals diagnosed with reportedly sporadic breast cancer. UM was sampled distant from PT site and confirmed for normal histology by pathologists. After DNA extraction SNP arrays were used to identify copy number alteration patterns (CNA) and copy number neutral loss-of-heterozygosity (LOH) in matched UM and PT samples. Next, targeted DNA sequencing was performed on sets of UM, PT and BL samples to identify somatic sequence variants of moderate frequency. Duplex sequencing of UM, PT, BL and SK samples of four selected individuals was used to screen for low-frequency somatic variants of *TP53* and *PIK3CA* genes in the normal mammary gland tissue.

Supplementary Figure 2

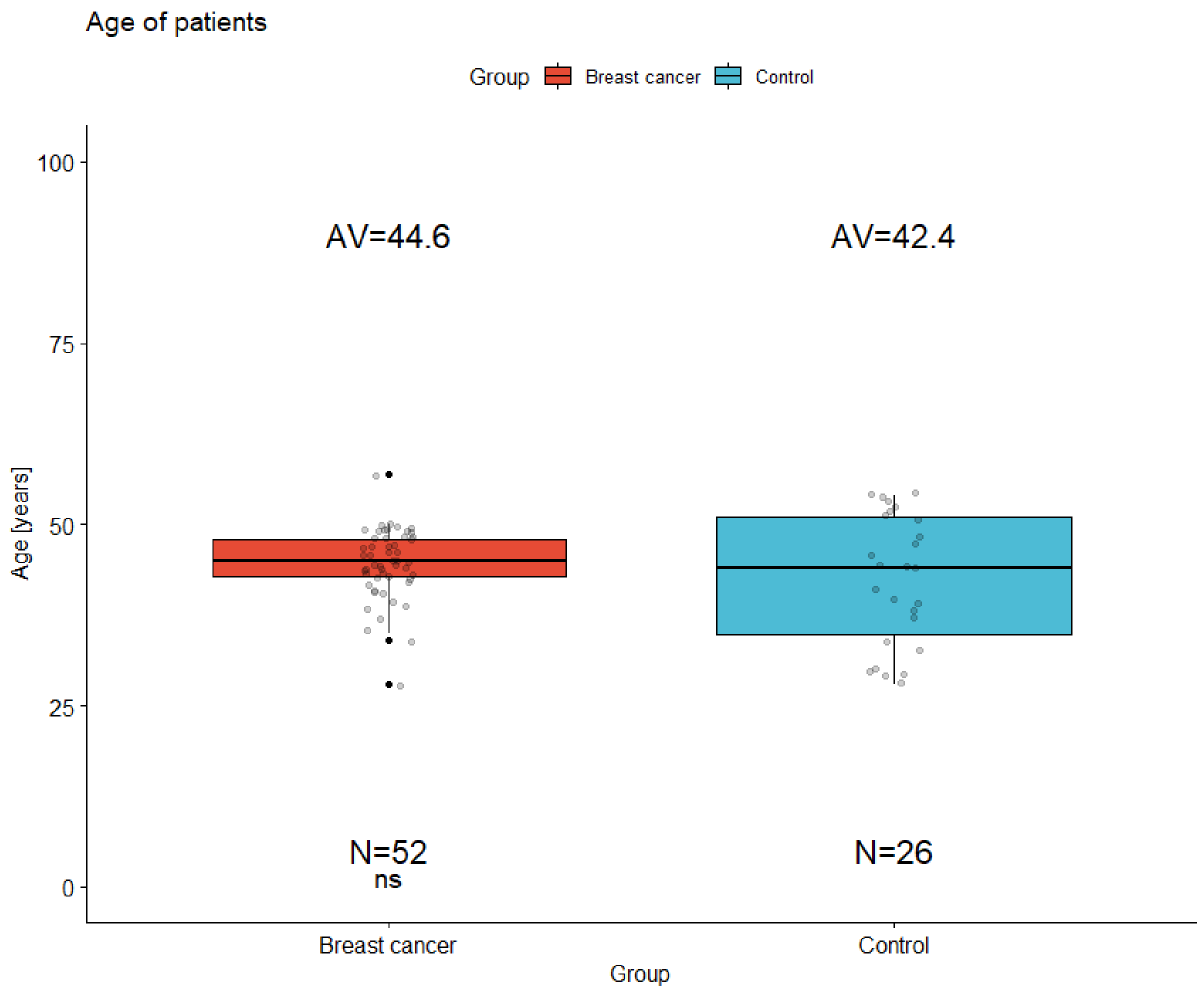

Supplementary Figure 2. Age matching and distribution in the breast cancer patient cohort and the control group. Boxplots show the total number (N) of patients, average age (AV) and age range in the healthy (blue) and breast cancer (red) groups. The difference in age between groups was tested with the Mann-Whitney U test. ns – not statistically significant

Supplementary Figure 3

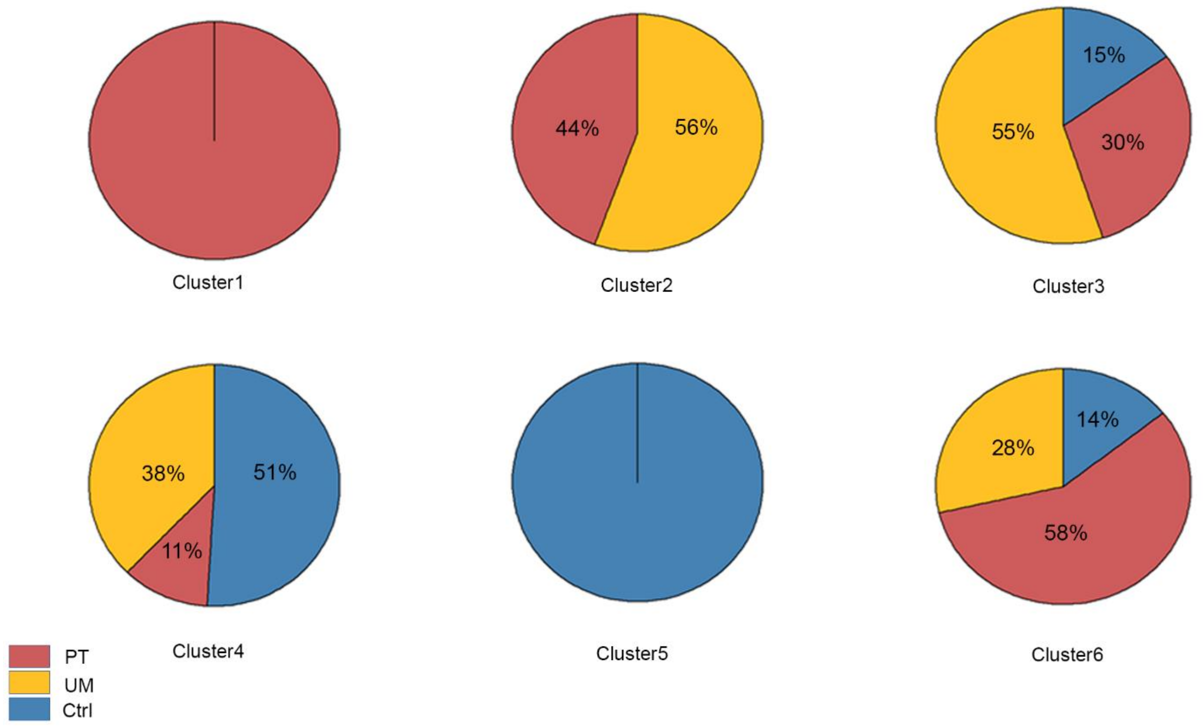

Supplementary Figure 3. Pie charts with uninvolved margin (UM), tumor (PT) and control (Ctrl) sample proportions within clusters 1-6 identified by hierarchical clustering (Figure 1). Tissue sample counts within the identified clusters: Cluster 1 – 19 PT samples; Cluster 2 – 8 PT samples, 10 UM samples; Cluster 3 – 6 PT samples, 11 UM samples, 3 control samples; Cluster 4 – 7 PT samples, 20 UM samples, 27 control samples; Cluster 5 – 19 control samples; Cluster 6 – 3 PT samples, 2 UM sample and 1 control sample.

Supplementary Figure 4

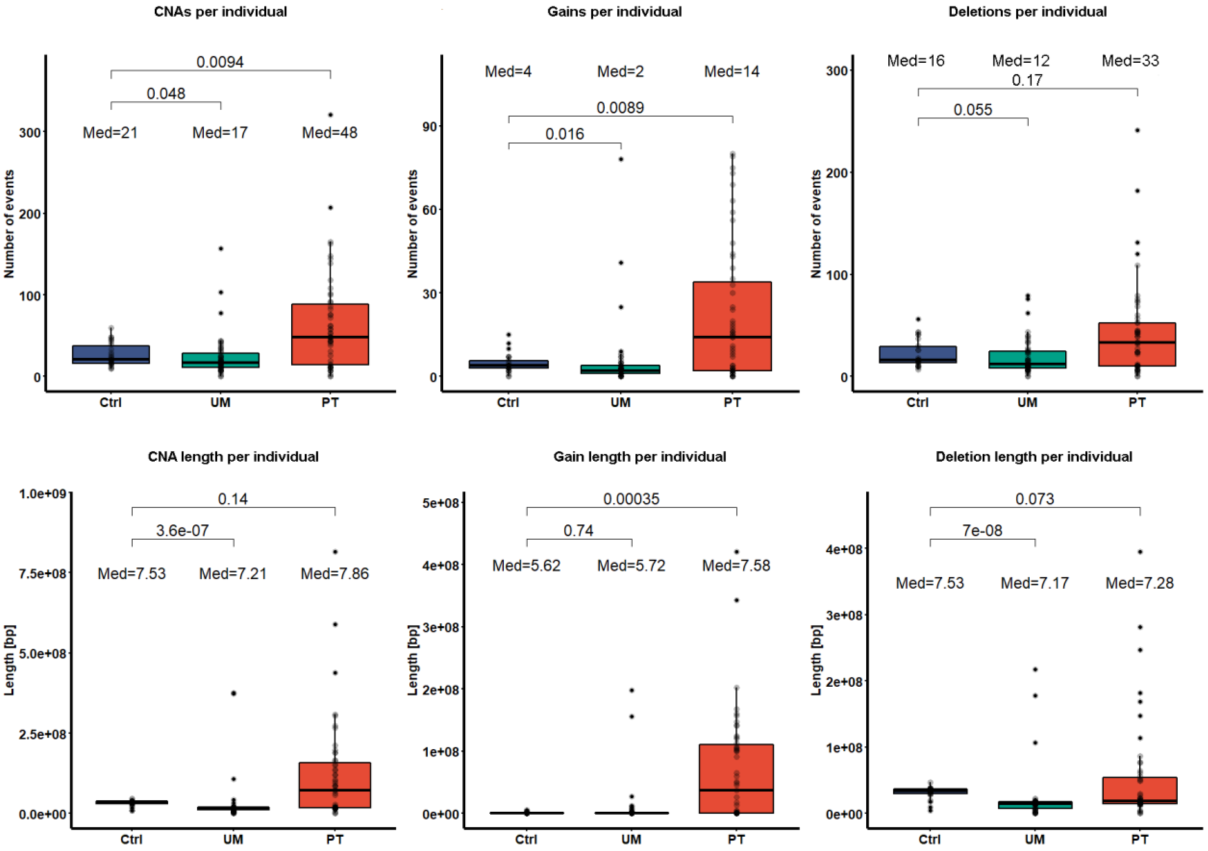

Supplementary Figure 4. Summary of number and size of Copy Number Alterations (CNAs) in tissues from breast cancer patients and the control cohort. Mammary gland samples from age-matched patients that underwent breast reduction surgery served as the control group (Supplementary Figure S2). CNA analysis was performed to compare the number and size of the detected changes between control samples and breast cancer patient tissues. Each data point represents calculations for a single patient. Statistical significance was tested with Wilcoxon test (p value is provided next to comparison). The median value of length size (Med) is presented as log10 (Y-axis)).

Supplementary Figure 5

a

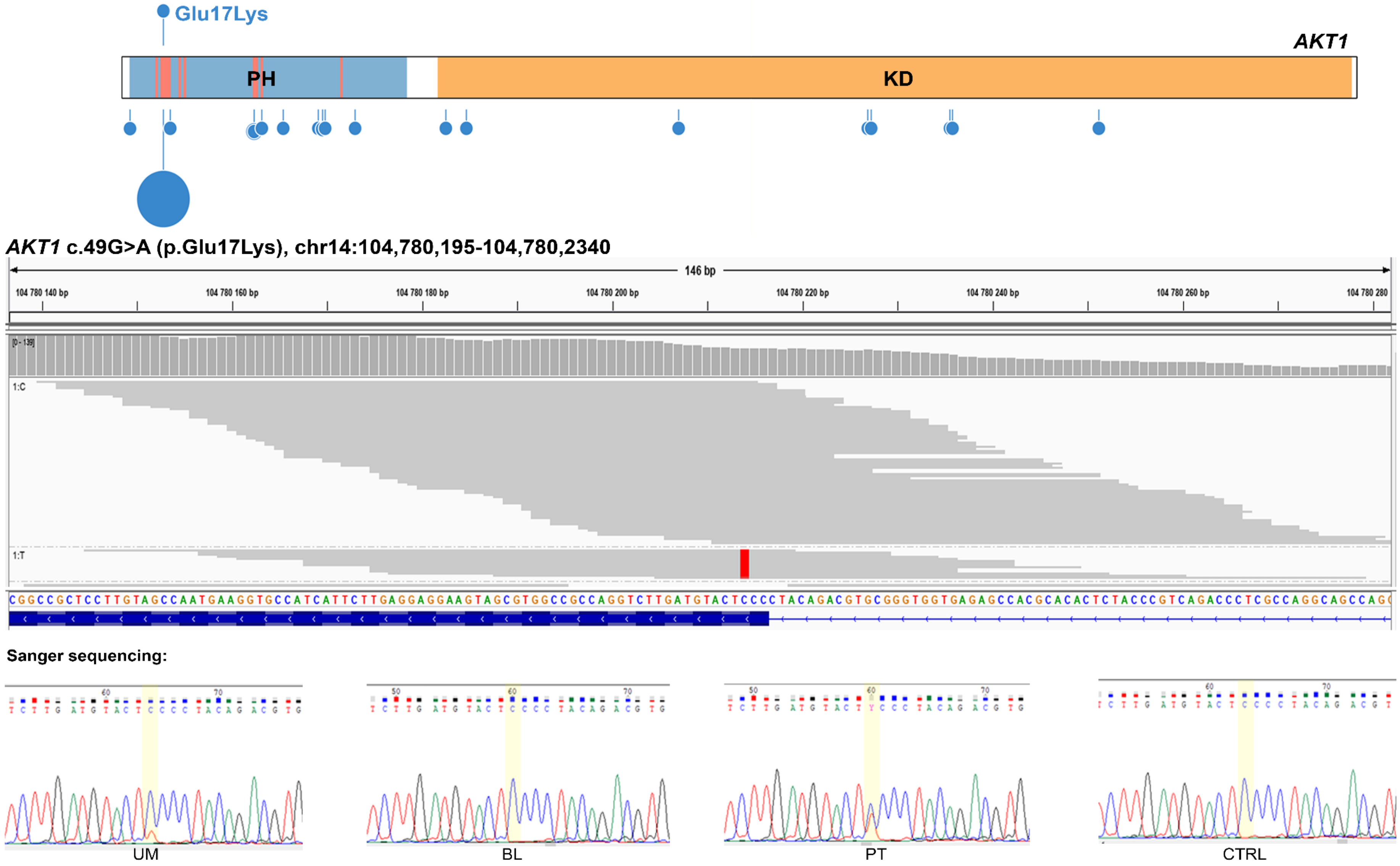

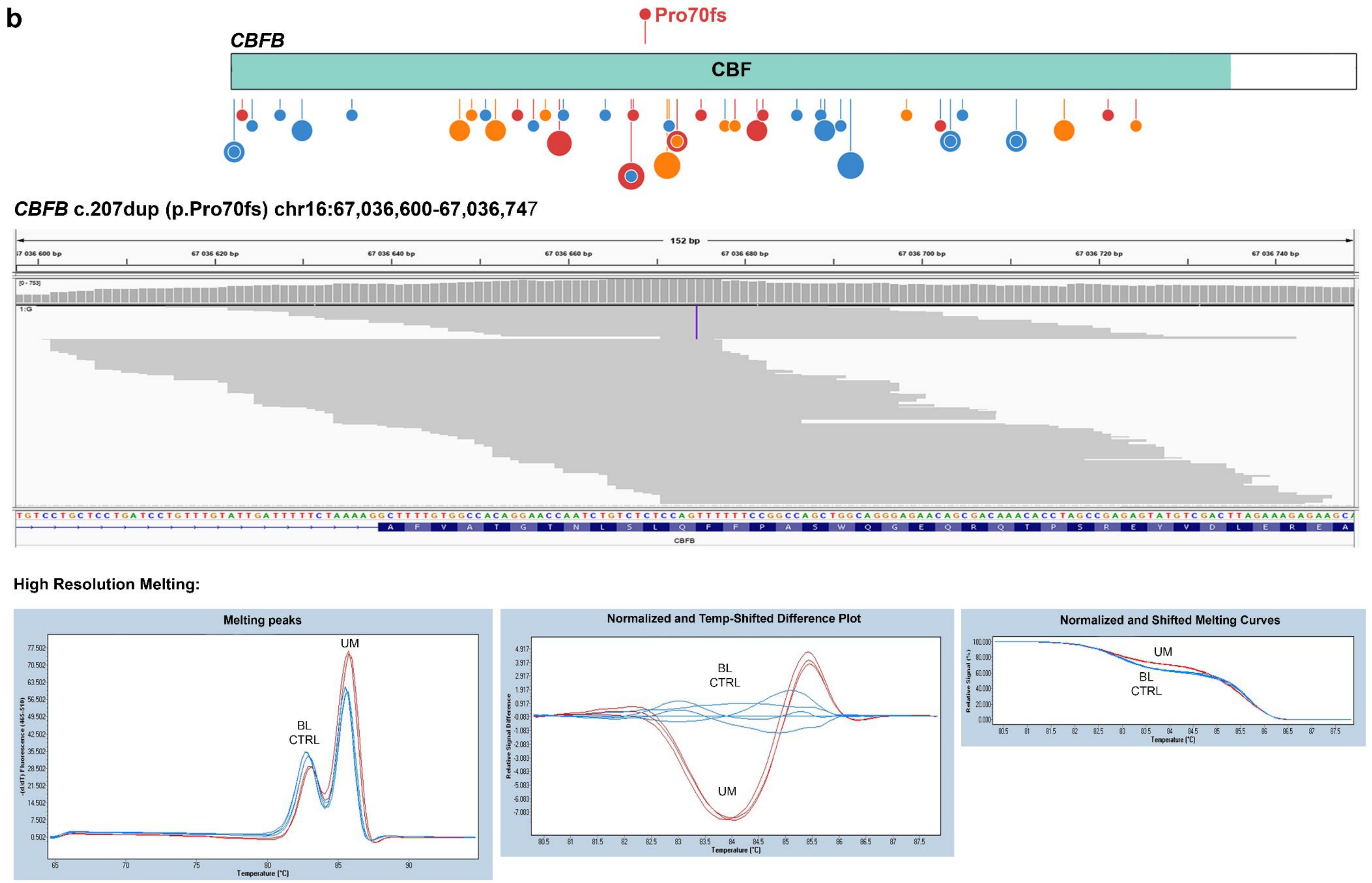
b
Pro70fs
CBFB
CBF
CBFB c.207dup (p.Pro70fs) chr16:67,036,600-67,036,747
152 bp
CBFB
High Resolution Melting:
Melting peaks
UM
BL
CTRL
Temperature (°C)
Normalized and Temp-Shifted Difference Plot
BL
CTRL
UM
Relative Signal Difference
Temperature (°C)
Normalized and Shifted Melting Curves
UM
BL
CTRL
Relative Signal (%)
Temperature (°C)

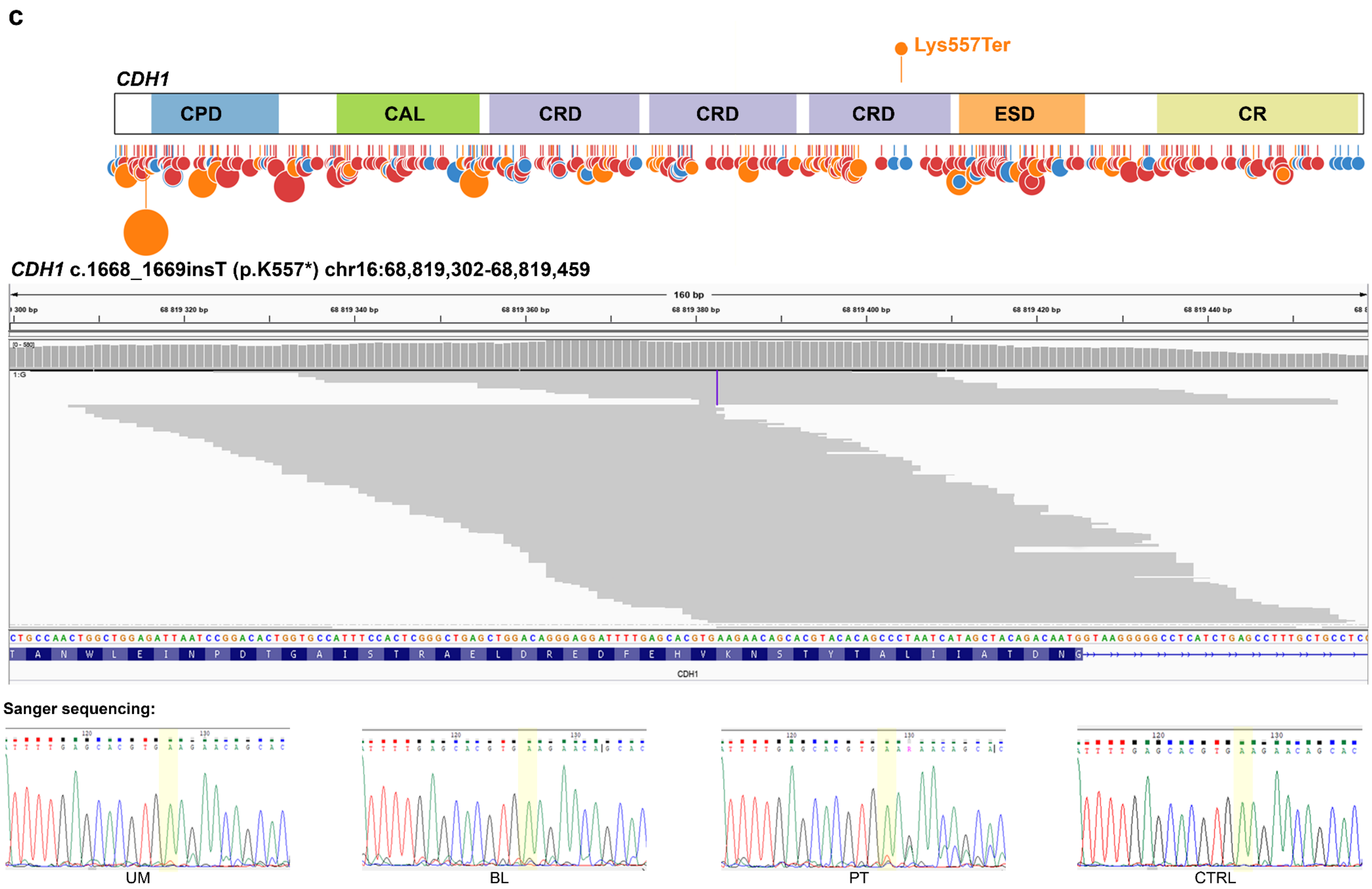
c
Lys557Ter
CDH1
CPD
CAL
CRD
CRD
CRD
ESD
CR
CDH1 c.1668_1669insT (p.K557*) chr16:68,819,302-68,819,459
160 bp
CDH1
Sanger sequencing:
UM
BL
PT
CTRL

d

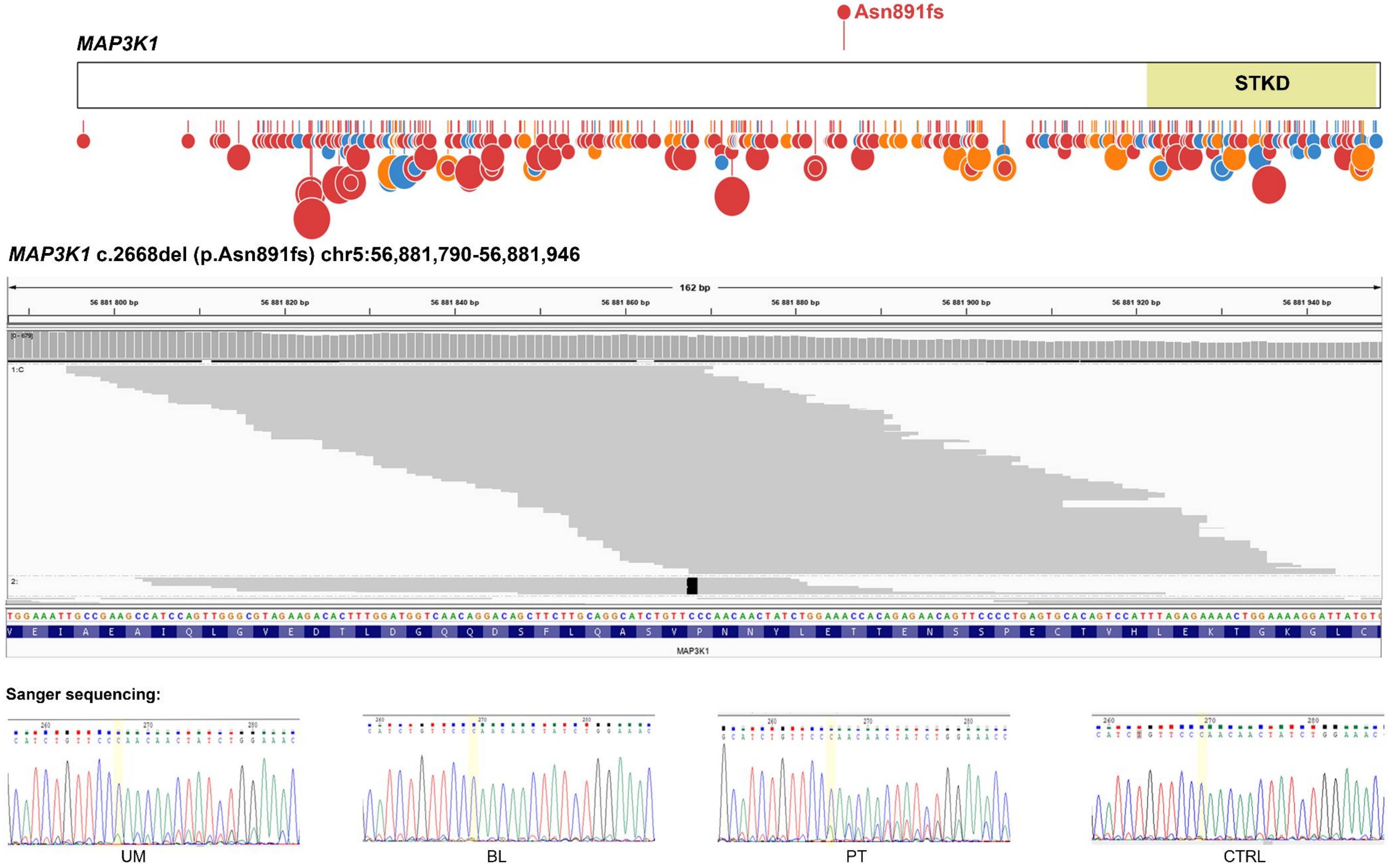
Asn891fs
MAP3K1
STKD
MAP3K1 c.2668del (p.Asn891fs) chr5:56,881,790-56,881,946
162 bp
MAP3K1
Sanger sequencing:
UM
BL
PT
CTRL

e

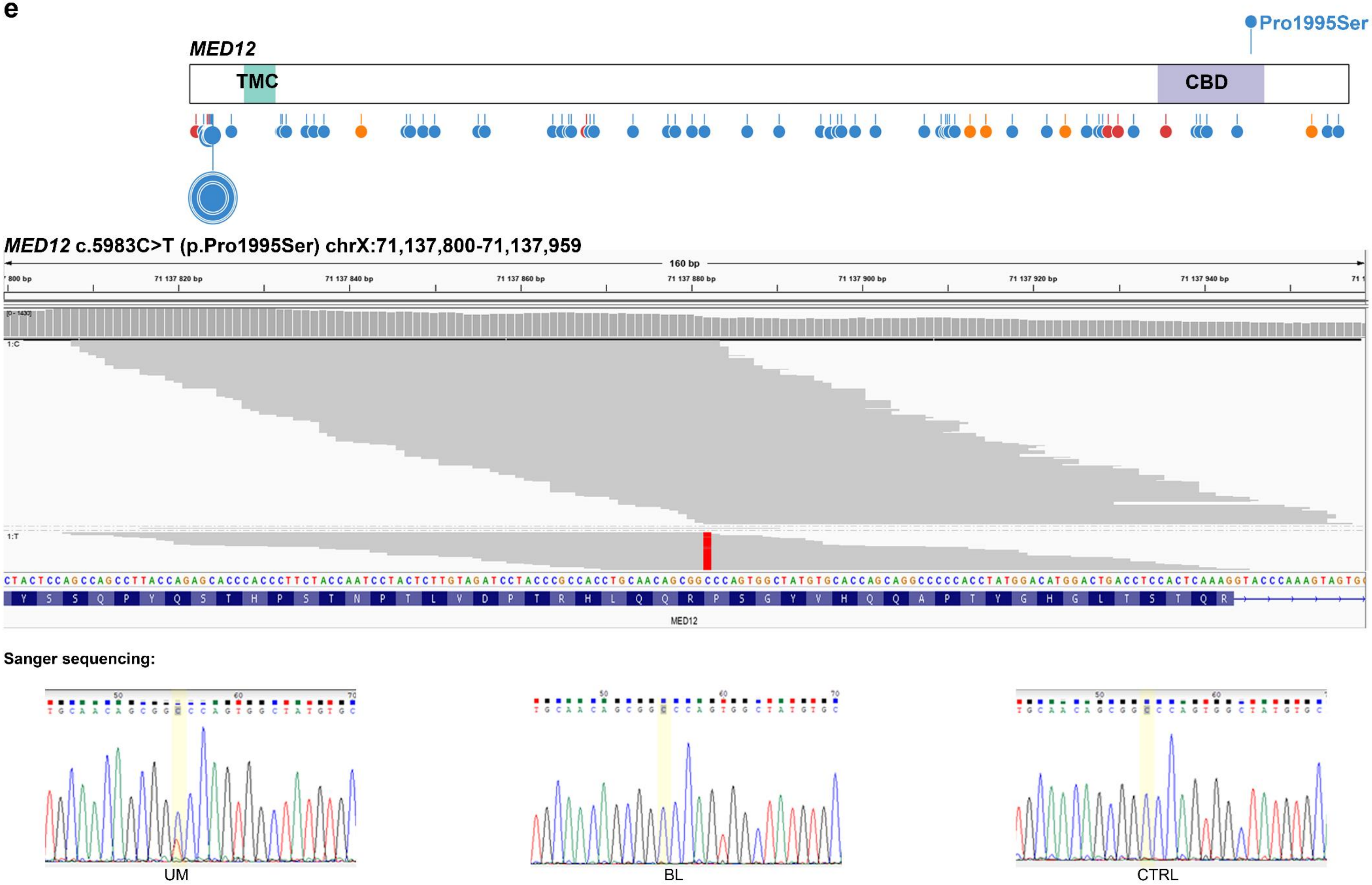
Pro1995Ser
MED12
TMC
CBD
MED12 c.5983C>T (p.Pro1995Ser) chrX:71,137,800-71,137,959
160 bp
MED12
Sanger sequencing:
UM
BL
CTRL

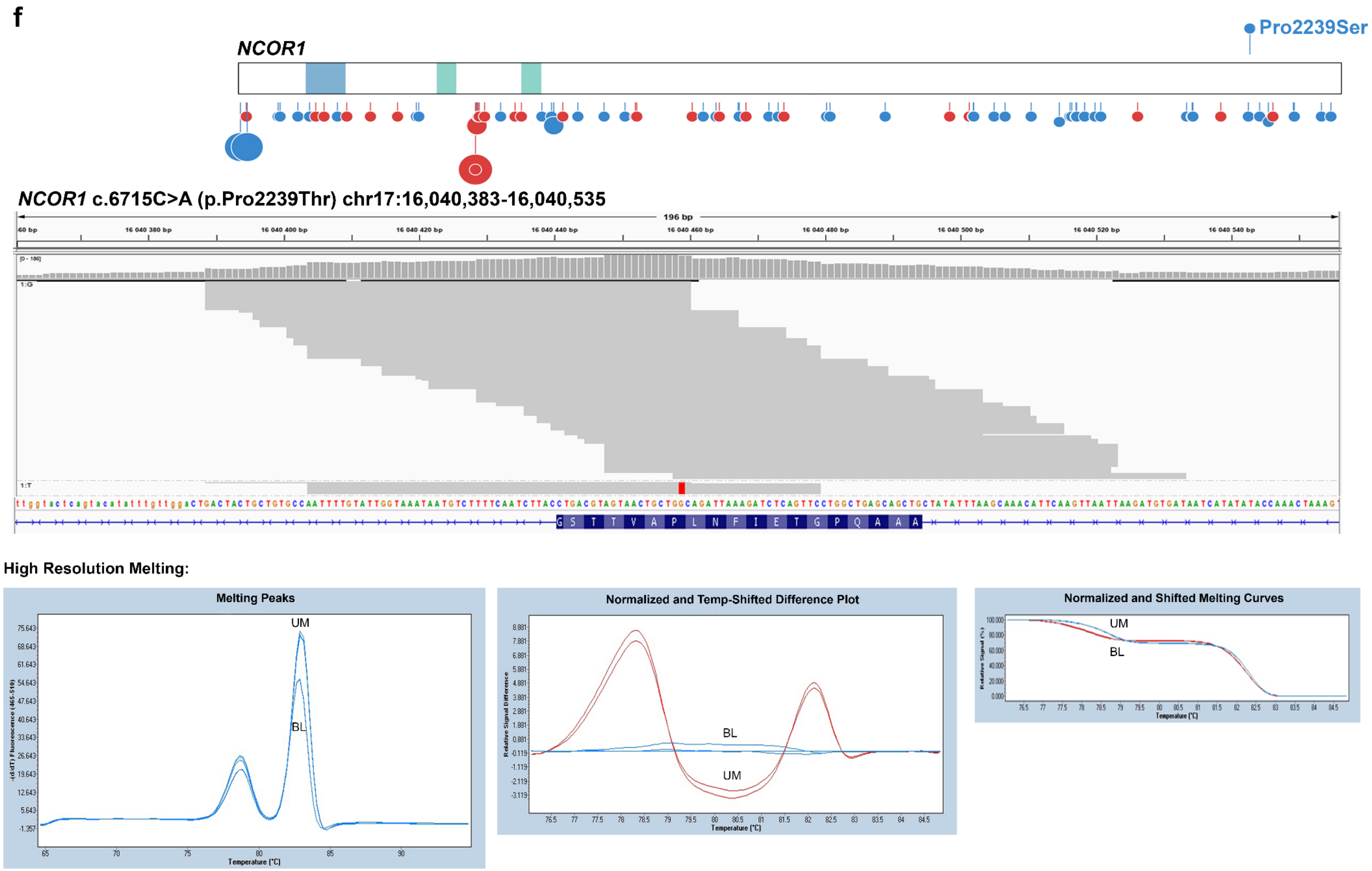

f
Pro2239Ser
NCOR1
NCOR1 c.6715C>A (p.Pro2239Thr) chr17:16,040,383-16,040,535
196 bp
High Resolution Melting:
Melting Peaks
UM
BL
Temperature (°C)
Normalized and Temp-Shifted Difference Plot
BL
UM
Relative Signal Difference
Temperature (°C)
Normalized and Shifted Melting Curves
UM
BL
Relative Signal (%)
Temperature (°C)

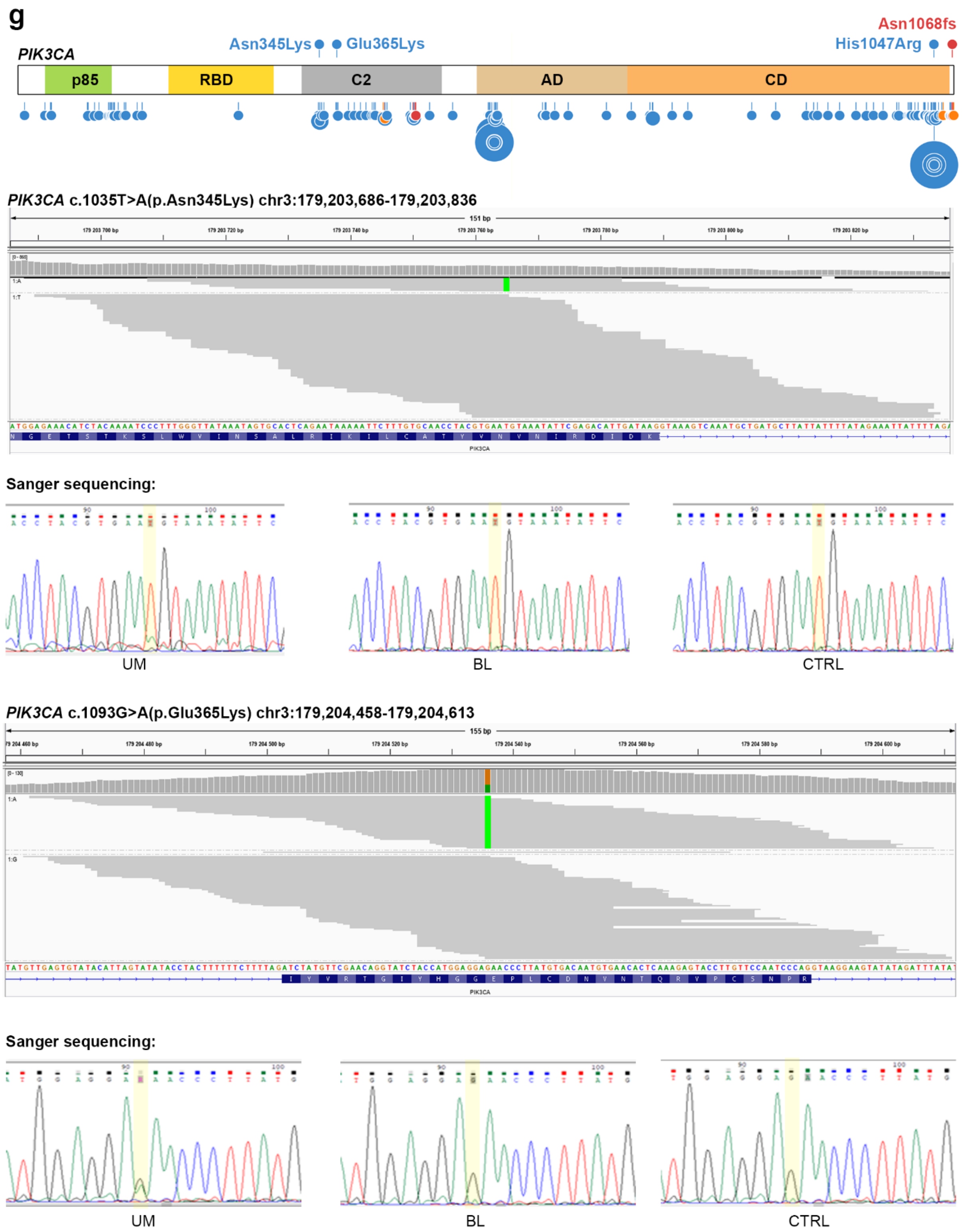
g
PIK3CA
Asn345Lys
Glu365Lys
Asn1068fs
His1047Arg
p85
RBD
C2
AD
CD
PIK3CA c.1035T>A(p.Asn345Lys) chr3:179,203,686-179,203,836
151 bp
Sanger sequencing:
UM
BL
CTRL
PIK3CA c.1093G>A(p.Glu365Lys) chr3:179,204,458-179,204,613
155 bp
Sanger sequencing:
UM
BL
CTRL

***PIK3CA* c.3140A>G(p.His1047Arg) chr3:179,234,222-179,234,373**

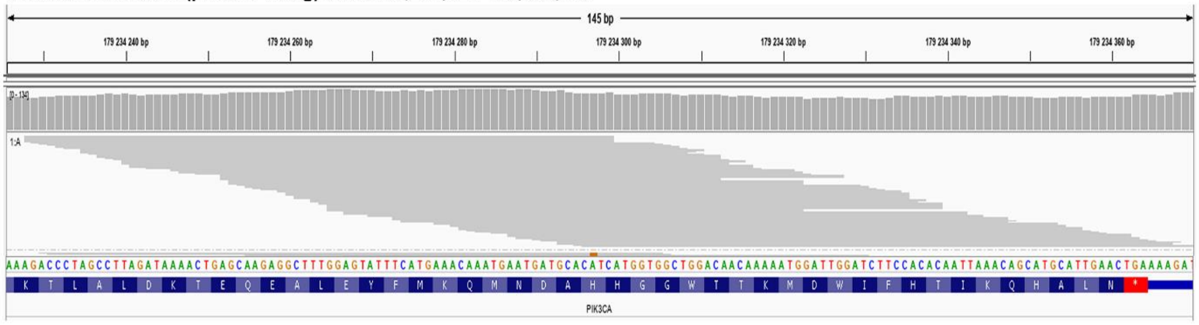

Sanger sequencing:

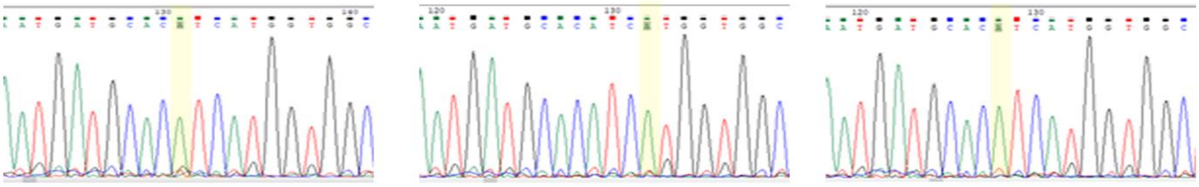

UM BL CTRL

***PIK3CA* c.3202dup(p.Asn1068fs) chr3:179,234,282-179,234,436**

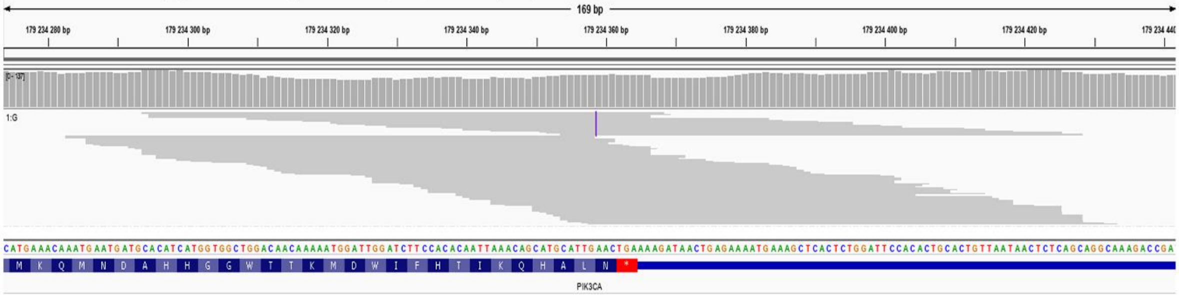

Sanger sequencing:

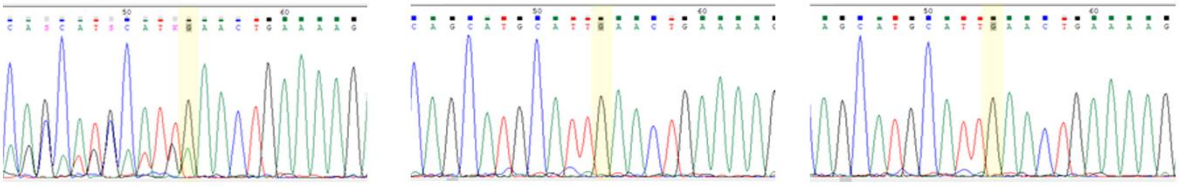

UM BL CTRL

h

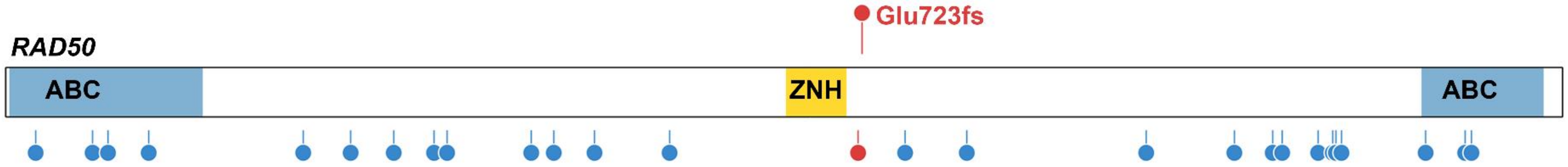

***RAD50* c.2165dup (p.Glu723fs) chr5:132,595,682-132,595,837**

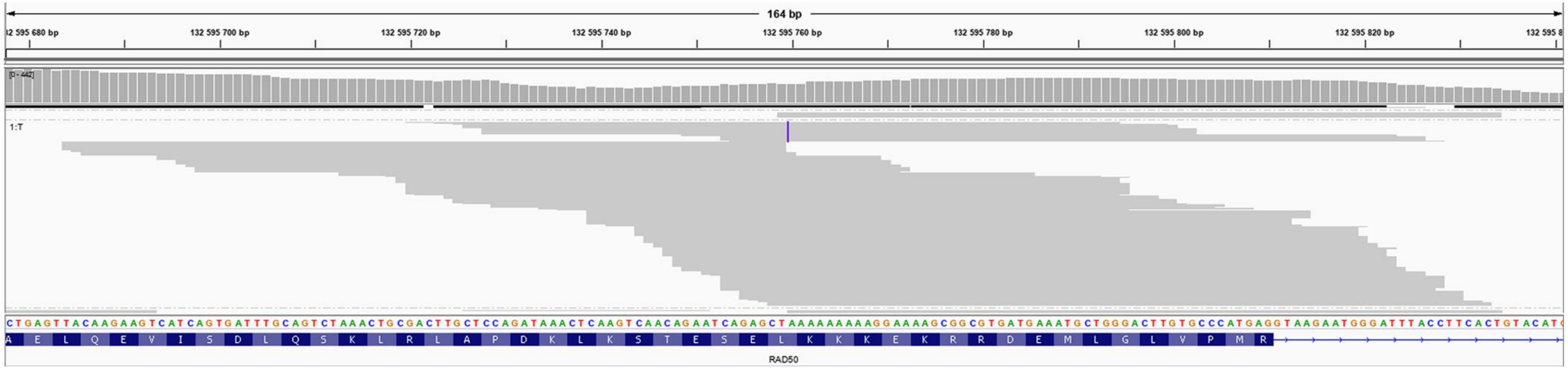

High Resolution Melting:

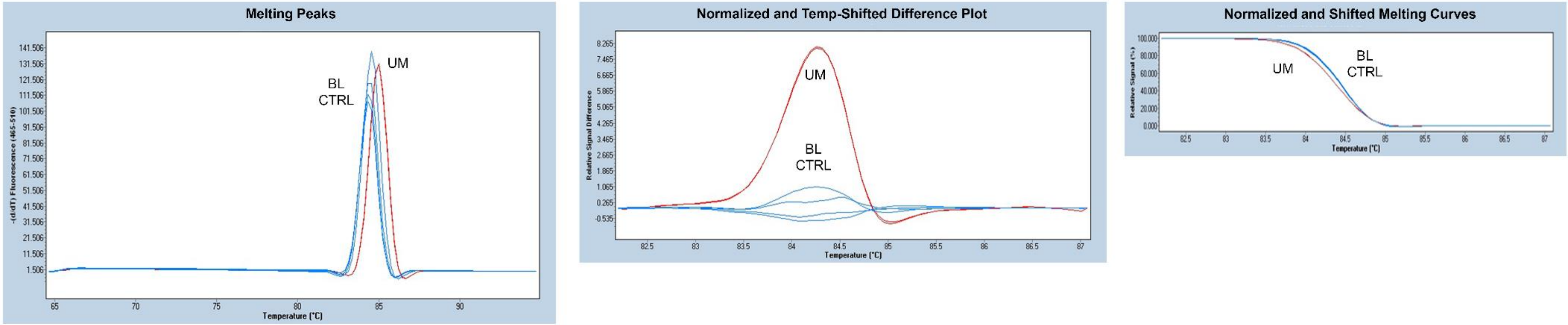

i

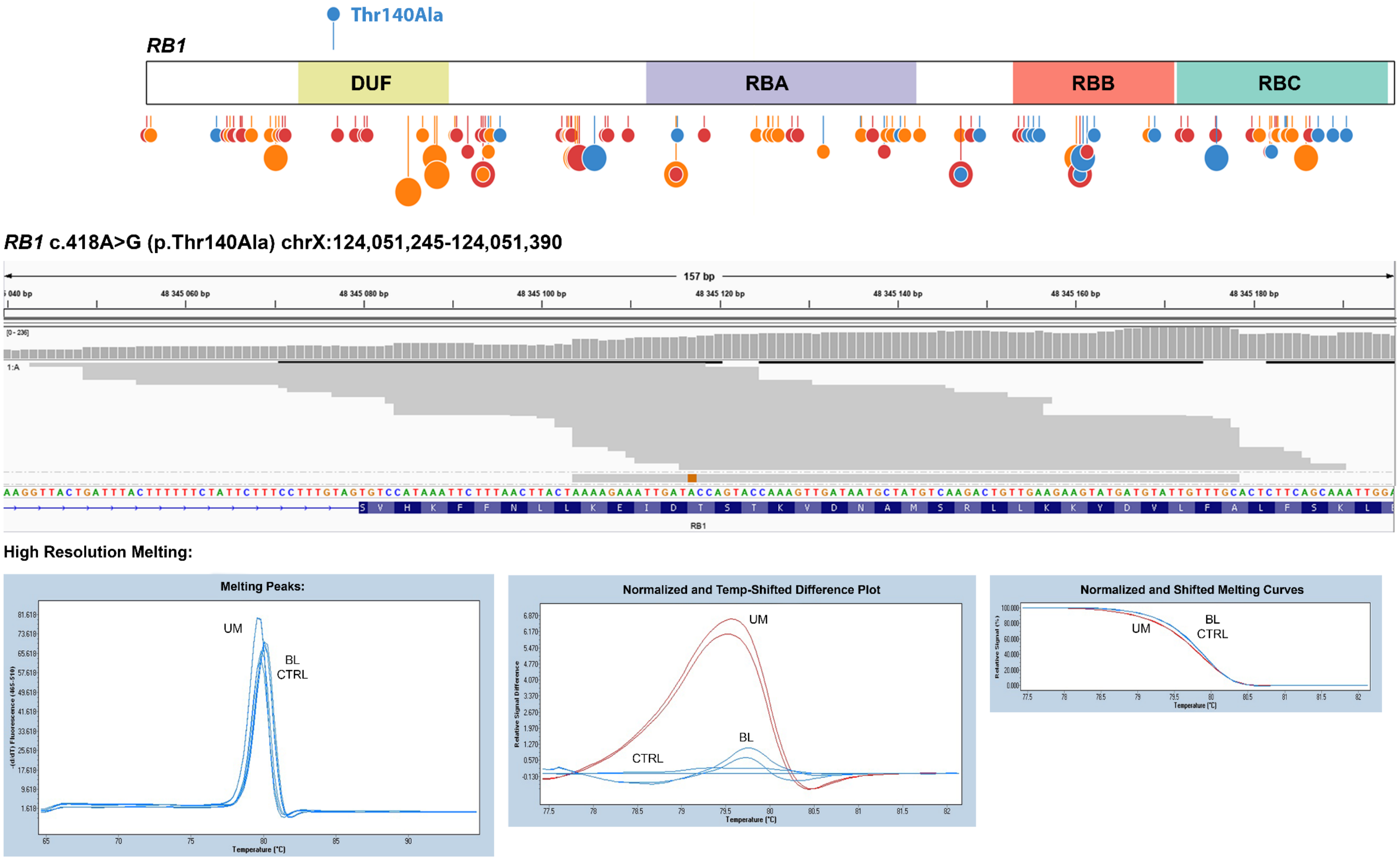
Thr140Ala
RB1
DUF
RBA
RBB
RBC
RB1 c.418A>G (p.Thr140Ala) chrX:124,051,245-124,051,390
157 bp
RB1
High Resolution Melting:
Melting Peaks:
UM
BL
CTRL
Temperature (°C)
Normalized and Temp-Shifted Difference Plot
UM
CTRL
BL
Temperature (°C)
Normalized and Shifted Melting Curves
UM
BL
CTRL
Temperature (°C)

j

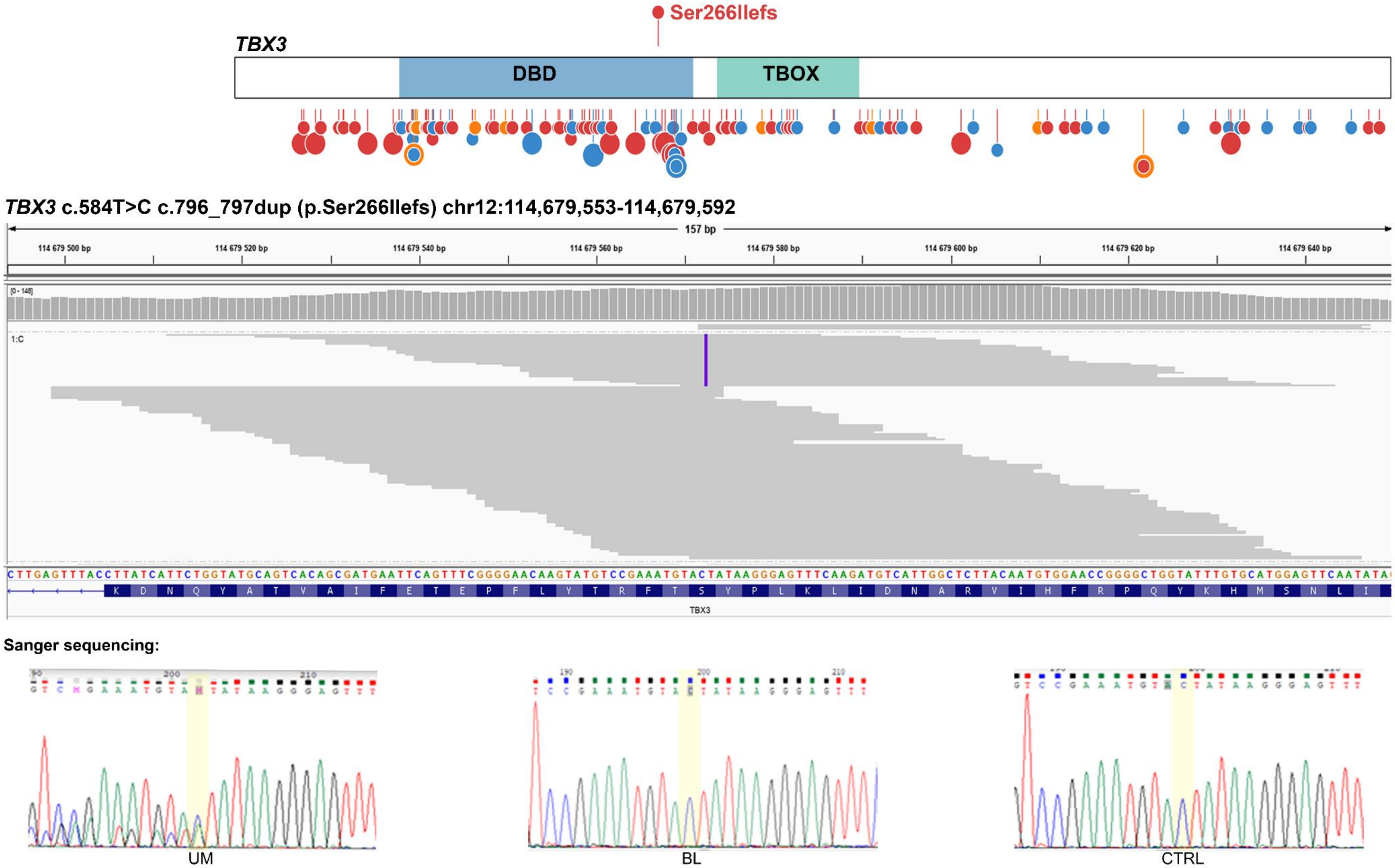

Ser266Ilefs
TBX3
DBD
TBOX
TBX3 c.584T>C c.796_797dup (p.Ser266Ilefs) chr12:114,679,553-114,679,592
157 bp
114 679 500 bp
114 679 520 bp
114 679 540 bp
114 679 560 bp
114 679 580 bp
114 679 600 bp
114 679 620 bp
114 679 640 bp
TBX3
Sanger sequencing:
UM
BL
CTRL

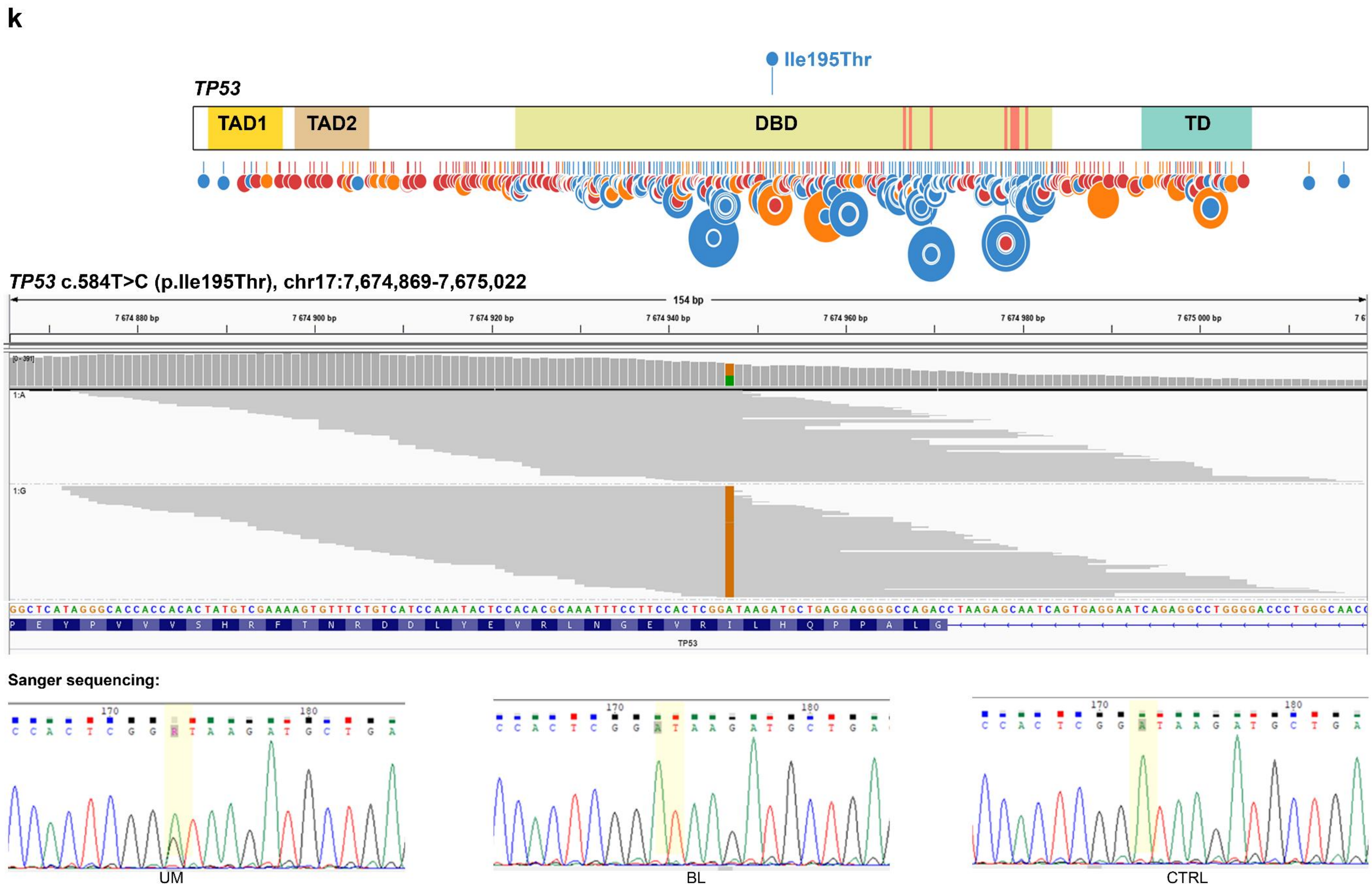
k
Ile195Thr
TP53
TAD1
TAD2
DBD
TD
TP53 c.584T>C (p.Ile195Thr), chr17:7,674,869-7,675,022
154 bp
7 674 880 bp
7 674 900 bp
7 674 920 bp
7 674 940 bp
7 674 960 bp
7 674 980 bp
7 675 000 bp
TP53
Sanger sequencing:
UM
BL
CTRL

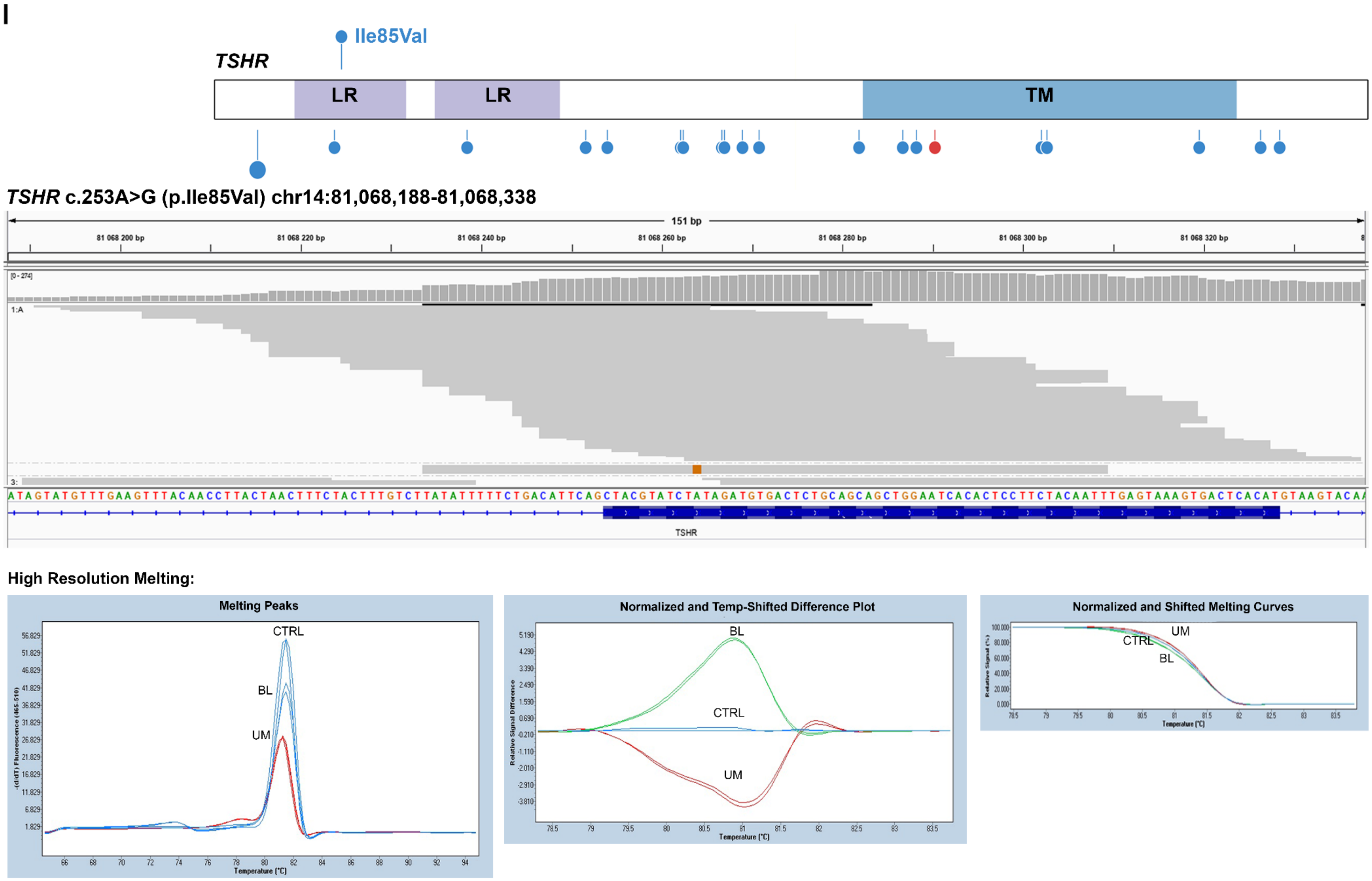
I
Ile85Val
TSHR
LR
LR
TM
TSHR c.253A>G (p.Ile85Val) chr14:81,068,188-81,068,338
151 bp
TSHR
High Resolution Melting:
Melting Peaks
CTRL
BL
UM
Temperature (°C)
Normalized and Temp-Shifted Difference Plot
BL
CTRL
UM
Relative Signal Difference
Temperature (°C)
Normalized and Shifted Melting Curves
UM
CTRL
BL
Temperature (°C)

Supplementary Figure 5. Somatic DNA variants detected in the normal mammary gland samples of sporadic breast cancer patients. Upper part of the lollipop plots present predicted amino acid change caused by the detected variant. Lower part presents somatic variants detected in the breast tumor samples, reported in the COSMIC database (https://cancer.sanger.ac.uk/cosmic). Missense variants are represented by blue dots, frameshift variants by red dots and nonsense by orange dots. Lollipop plots were prepared based on images generated with the ProteinPaint application[52]. Middle panels present aligned reads from targeted DNA sequencing of UM samples with marked variant reads and were prepared based on IGV (Integrative Genomics Viewer, http://www.broadinstitute.org/igv). Lower panels include variant confirmation by Sanger sequencing or High Resolution Melting (HRM). UM – uninvolved mammary gland, PT – primary tumor, BL – peripheral blood, CTRL – peripheral blood from an unrelated individual. (a) Presentation of *AKT1* c.49G>A variant. PH (Protein Kinase B-like pleckstrin homology domain), red lines within the PH domain indicate phosphoinositide binding sites; KD (Catalytic domain of the Serine/Threonine Kinase). (b) Presentation of *CBFB* c.207dup variant. CBF (Core binding factor beta subunit). (c) Presentation of *CDH1* c.1668_1669insT variant. CPD (cadherin prodomain); CAL (cadherin repeat-like domain); CRD (cadherin repeat domain); ESD (early set domain); CR (cytoplasmic region). (d) Presentation of *MAP3K1* c.2668del. STKD (Serine/Threonine protein kinase catalytic domain). (e) Presentation of *MED12* c.5983C>T variant. TMC (transcription mediator complex subunit); CBD (Catenin-binding domain). (f) Presentation of *NCOR1* c.6715C>A variant. GPS2 (G-protein pathway suppressor 2-interacting domain); SANT (SWI3, ADA2, N-CoR and TFIIIB DNA-binding domains). (g) Presentation of *PIK3CA* somatic variants: c.1035T>A, c.1093G>A, c.3140A>G and c.3203dup detected in the normal mammary gland samples. p85 (p85-binding domain); RBD (Ras-binding domain); C2 (C2 domain); AD (accessory domain); CD (catalytic domain). (h) Presentation of *RAD50* c.2165dup variant. ABC (ATP-binding cassette), ZNH (zinc hook motif). (i) Presentation of *RB1* c.418A>G variant. RBA (Retinoblastoma-associated protein A domain); RBB (Retinoblastoma-associated protein B domain); RBC (Retinoblastoma-associated protein C domain). (j) Presentation of *TBX3* c.796_797dup variant. DBD (DNA-binding domain); TBOX (T-box transcription factor domain). (k) Presentation of *TP53* c.584T>C variant. TAD1, TAD2 (transcription activation domain 1 and 2); DBD (DNA-binding domain), DNA-binding sites are marked with red lines; TD (tetramerization domain). (l) Presentation of *TSHR* c.253A>G variant. LR (leucine repeats); TM (transmembrane receptor domain).

Supplementary Figure 6

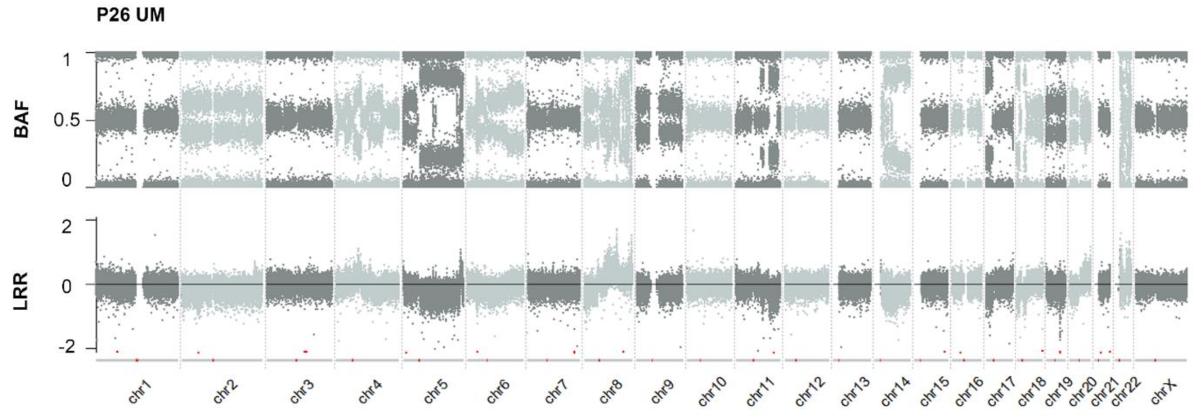

Supplementary Figure 6. Genomic destabilization as detected in the uninvolved mammary gland sample (UM) carrying somatic *AKT1* c.49G>A (p.Glu17Lys) variant. Plots present B allele frequency (BAF) and log R ratio (LRR) per chromosome.

Supplementary Figure 7

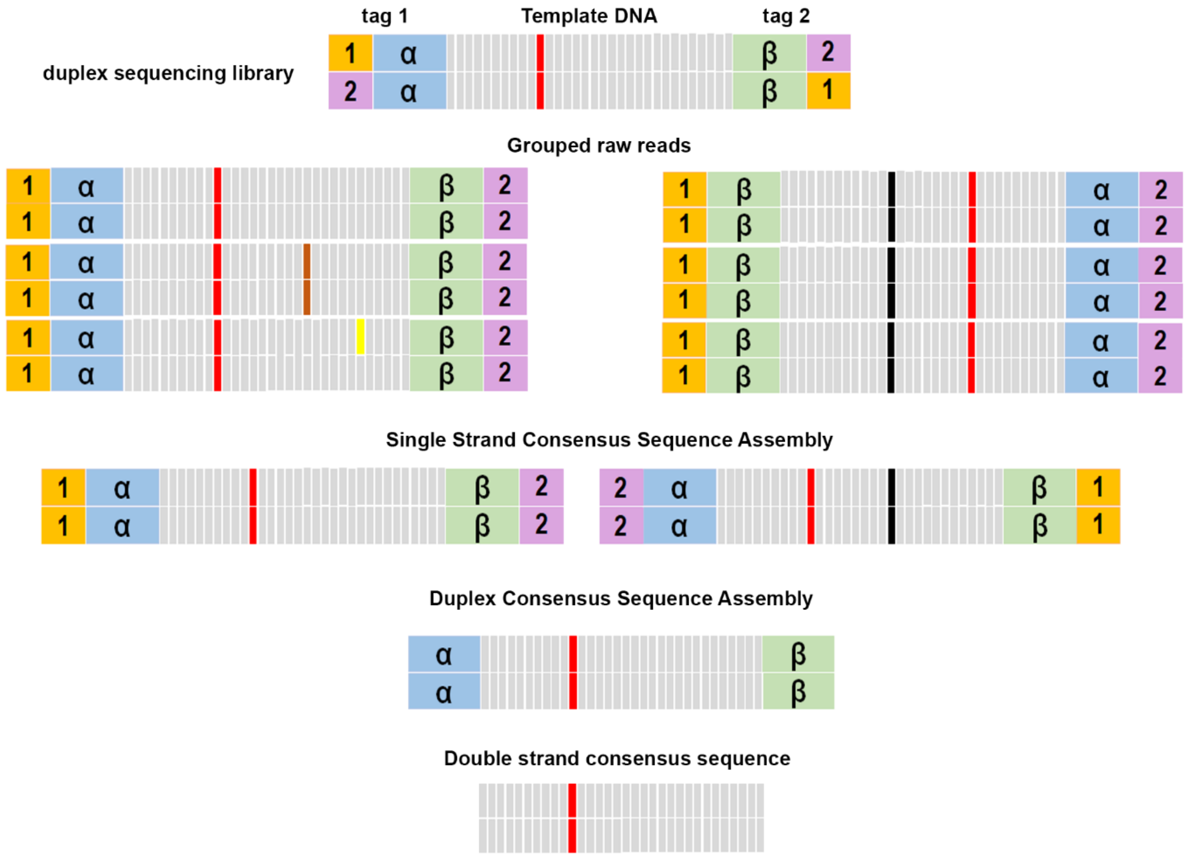

Supplementary Figure 7. Schematic presentation of variant detection by duplex sequencing. During library preparation each DNA fragment is ligated to adapters with α and β duplex tags and flow cell sequences 1 and 2. After amplification two types of PCR products (αβ and βα) are grouped into families. True variants that are present on both DNA strands appear in the majority (>66%) of family pair members (red). Artifactual mutations are identified after error correction when single-strand consensus sequences are obtained (brown, yellow and black). Duplex consensus sequence identifies true variants (red) by pairing the respective complementary single strand consensus sequences. This figure was prepared based on: https://commons.wikimedia.org/wiki/File:Duplex_sequencing_overview_alphabeta_fix.svg.

Supplementary Figure 8

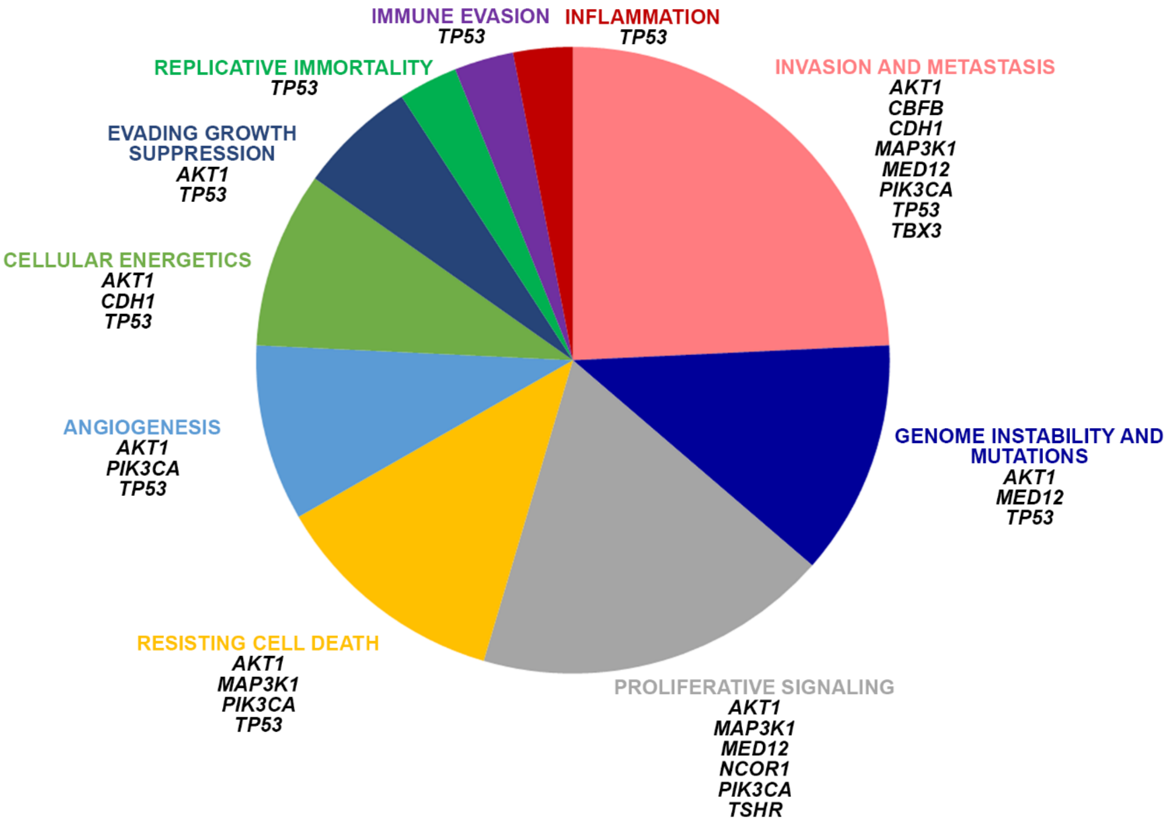

Supplementary Figure 8. Somatic mutations in UM samples and the hallmarks of cancer. The identified somatic variants of known breast cancer-associated genes are involved in the regulation of key processes that promote tumor growth and invasion: the hallmarks of cancer. The pie chart represents functional annotation of breast cancer-associated genes targeted by somatic mutations detected in the uninvolved mammary gland, according to the COSMIC database (https://cancer.sanger.ac.uk/cosmic) and the PANTHER classification system (http://www.pantherdb.org/).

Supplementary Figure 9

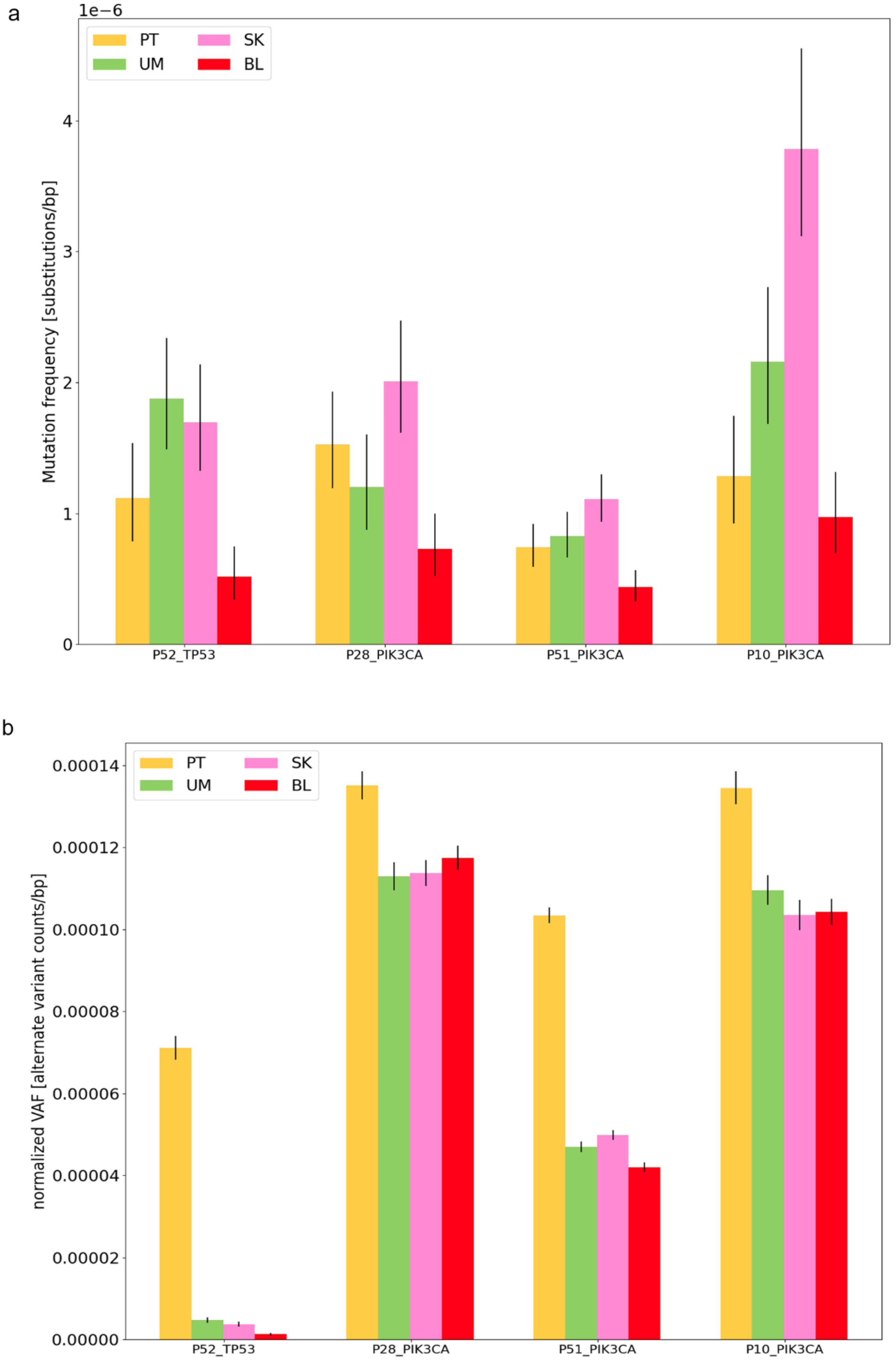

Supplementary Figure 9. (a) Mutation frequency of *PIK3CA* and *TP53* detected by duplex sequencing in primary tumor (PT), uninvolved mammary gland (UM), blood (BL) and skin (SK) samples of 4 individuals (P52, P28, P51, P10). Each bar represents the mutation frequency per tissue estimated as the sum of the different substitutions divided by the total number of sequenced base pairs (average gene coverage times the size of the gene). **(b)** Normalized variant allele frequency (alternate allele count divided by the total number of sequenced base pairs) measured in 4 tissues in 4 individuals (P52, P28, P51, P10). The error bars are calculated as the 95% confidence intervals of a Poisson distribution.

Supplementary Figure 10

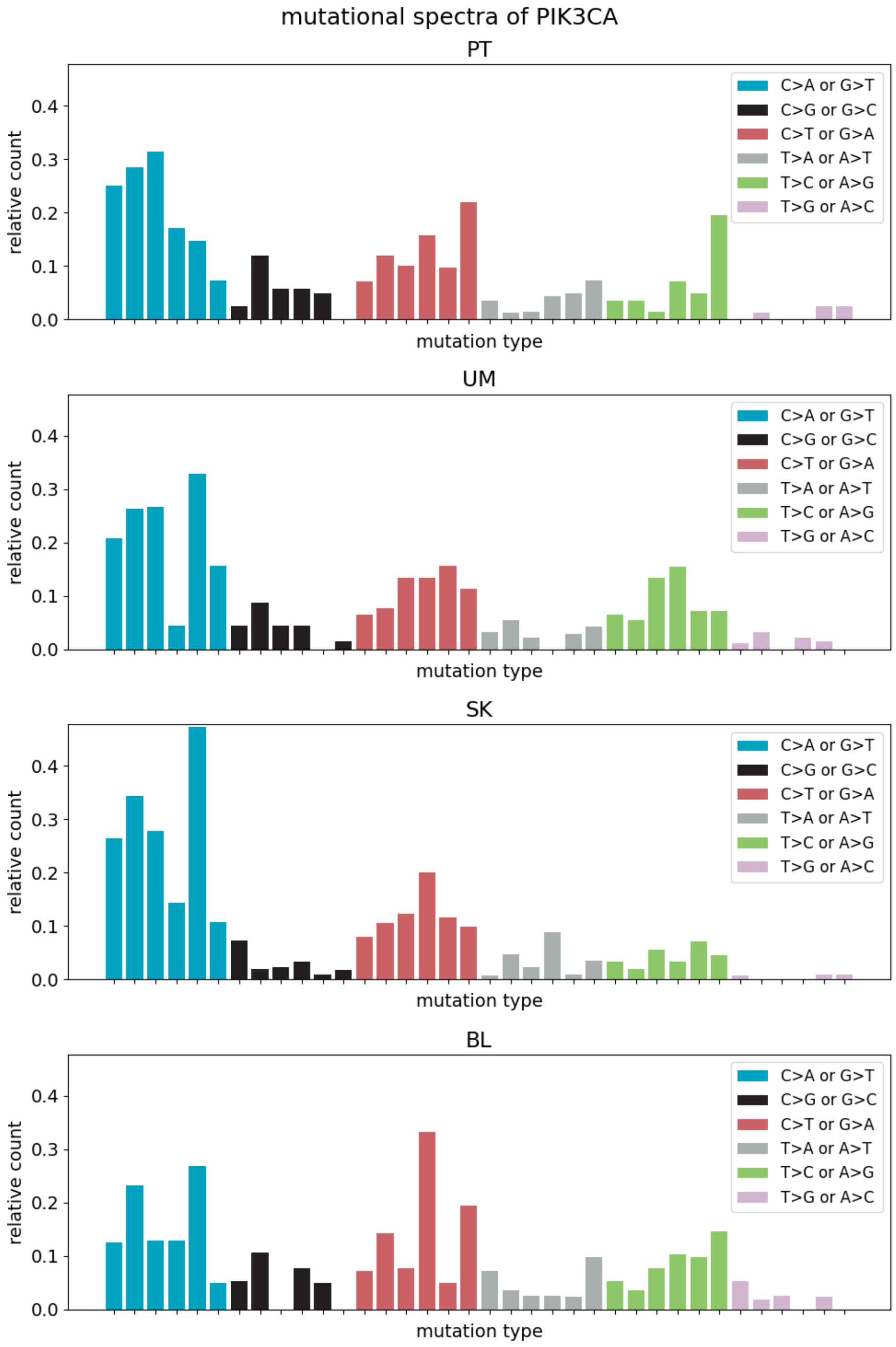

Supplementary Figure 10. Mutational spectra of *PIK3CA* variants. The relative count per substitution type is shown for all variants detected by duplex sequencing present in primary tumor (PT), uninvolved mammary gland (UM), blood (BL) and skin (SK) samples. The relative count of 3 individuals is shown per substitution type (ordered as P51 with 84 variants in PT, 91 variants in UM, 151 variants in SK and 56 variants in BL; P28 with 70 variants in PT, 45 variants in UM, 90 variants in SK and 39 variants in BL; P10 with 41 variants in PT, 70 variants in UM, 112 variants in SK and 41 variants in BL).
