# Supplementary material for: High prevalence of somatic PIK3CA and TP53 pathogenic variants in the normal mammary gland tissue of sporadic breast cancer patients revealed by duplex sequencing
Source: NPJ Breast Cancer. 2022 Jun 29;8:76. doi: 10.1038/s41523-022-00443-9 (PMC9243094; doi:10.1038/s41523-022-00443-9)
Supplement: Supplementary file 8 — Supplementary Table 7 [file 41523_2022_443_MOESM8_ESM.docx]

| **Gene** | **Gene function evidence in the context of cancer** |
| --- | --- |
| *AKT1* | Constitutively active Akt1 negatively regulates DNA repair processes, creating a BRCA1-deficient phenotype without *BRCA1* mutations^1^. *AKT1* c.49G>A (p.Glu17Lys) was reported in 3% of primary breast cancers, exclusively ER-positive^2^. Notably, the UM sample with the c.49G>A variant had also an excessive load of DNA copy number alterations, reflecting the deleterious effect of this variant on the genome^1^ (Supplementary Figure 6). |
| *CBFB* | Forms a transcriptional complex with RUNX1 and is involved in the regulation of translation^3^. Inactivating pathogenic variants have been associated with ER+ and lower grade tumors^4^. |
| *CDH1* | Tumor suppressor, regulates cell adhesion, prevents invasion and metastasis. Truncating variants are common in breast tumors and associated with the lobular histological type^5^. |
| *MAP3K1* | Involved in regulation of proliferation, growth and apoptosis, loss of MAP3K1 can render cells insensitive to death-inducing signals^6^. |
| *MED12* | Regulates RNA polymerase II transcription and gene expression^7^. MED12 regulates signal transduction through the Wnt/β-catenin pathway through direct interactions with β-catenin^8^. *MED12* pathogenic variants were observed in malignant phyllodes tumors and fibroadenomas with a greater frequency in the latter. Pareja et al., described *MED12* pathogenic variants as early events and when followed by additional activation of oncogenes or inactivation of suppressors can cause malignant progression^9^. |
| *NCOR1* | One of the most frequently mutated genes in breast tumors, element of the chromatin remodeling complex found to repress ER-mediated transcription^10^. |
| *RAD50* | Involved in DNA damage response as a part of the Mre11-Rad50-Nbs1 complex^11^. Germline *RAD50* c.2165dup p.Glu723fs variant was reported in patients with early-onset familial breast cancer^12^. |
| *RB1* | Tumor suppressor, coordinates cell cycle via several pathways, its loss deregulates cell cycle progression and is associated with genomic instability^13^. |
| *TBX3* | Regulates normal mammary gland development, truncating variants have been observed in breast tumors^4,10^. In ductal carcinoma *in situ* high *TBX3* expression is associated with progression from benign to invasive state^14^. |
| *TSHR* | Thyroid-stimulating hormone receptor involved in endocrine regulation of cell proliferation and differentiation. Thyroid hormone activity is associated with breast cancer risk. Breast cancer cell-based studies revealed thyroid hormone implication in ER regulation through MAPK kinases^15^. |

**Supplementary Table 7**. Summary of the function of breast cancer-associated genes targeted by somatic variants in the normal mammary gland (UM).

References:

1. Guirouilh-Barbat, J. K., Wilhelm, T. & Lopez, B. S. AKT1/BRCA1 in the control of homologous recombination and genetic stability: the missing link between hereditary and sporadic breast cancers. *Oncotarget* **1**, 691–699 (2010).

2. Yi, K. H., Axtmayer, J., Gustin, J. P., Rajpurohit, A. & Lauring, J. Functional analysis of non-hotspot AKT1 mutants found in human breast cancers identifies novel driver mutations: Implications for personalized medicine. *Oncotarget* **4**, 29–34 (2013).

3. Malik, N. et al. The transcription factor CBFB suppresses breast cancer through orchestrating translation and transcription. *Nat. Commun*. **10**, 1–15 (2019).

4. Pereira, B. et al. The somatic mutation profiles of 2,433 breast cancers refines their genomic and transcriptomic landscapes. *Nat. Commun*. **7**, 11479 (2016).

5. Christgen, M. et al. Lobular breast cancer: Clinical, molecular and morphological characteristics. *Pathol. Res. Pract*. **212**, 583–597 (2016).

6. Pham, T. T., Angus, S. P. & Johnson, G. L. MAP3K1: Genomic Alterations in Cancer and Function in Promoting Cell Survival or Apoptosis. *Genes and Cancer* **4**, 419–426 (2013).

7. Chang, H. Y. et al. MED12, TERT and RARA in fibroepithelial tumours of the breast. *J. Clin. Pathol*. **73**, 51–56 (2020).

8. Kim, S., Xu, X., Hecht, A. & Boyer, T. G. Mediator is a transducer of Wnt/β-catenin signaling. *J. Biol. Chem*. **281**, 14066–14075 (2006).

9. Pareja, F. et al. Phyllodes tumors with and without fibroadenoma-like areas display distinct genomic features and may evolve through distinct pathways. *npj Breast Cancer* **3**, 1–7 (2017).

10. Stephens, P. J. et al. The landscape of cancer genes and mutational processes in breast cancer. *Nature* **486**, 400–404 (2012).

11. Fagan-Solis, K. D. et al. A P53-Independent DNA Damage Response Suppresses Oncogenic Proliferation and Genome Instability. *Cell Rep*. **30**, 1385-1399.e7 (2020).

12. Lin, P. H. et al. Multiple gene sequencing for risk assessment in patients with early-onset or familial breast cancer. *Oncotarget* **7**, 8310–8320 (2016).

13. Witkiewicz, A. K. & Knudsen, E. S. Retinoblastoma tumor suppressor pathway in breast cancer: Prognosis, precision medicine, and therapeutic interventions. *Breast Cancer Res*. *16*, (2014).

14. Krstic, M. et al. TBX3 promotes progression of pre-invasive breast cancer cells by inducing EMT and directly up-regulating SLUG. *J. Pathol*. **248**, 191–203 (2019).

15. Liu, Y. C., Yeh, C. T. & Lin, K. H. Molecular functions of thyroid hormone signaling in regulation of cancer progression and anti-apoptosis. *Int. J. Mol. Sci*. **20**, 1–27 (2019).
